# Polysubstituted Pyridines from 1,4-Oxazinone Precursors

**DOI:** 10.1021/acs.joc.4c02389

**Published:** 2024-11-12

**Authors:** L. C. Thompson, Adrianne M. Kinsey, Zannatul Shahla, Jonathan R. Scheerer

**Affiliations:** Department of Chemistry, William & Mary, P.O. Box 8795, Williamsburg, Virginia 23187, United States

## Abstract

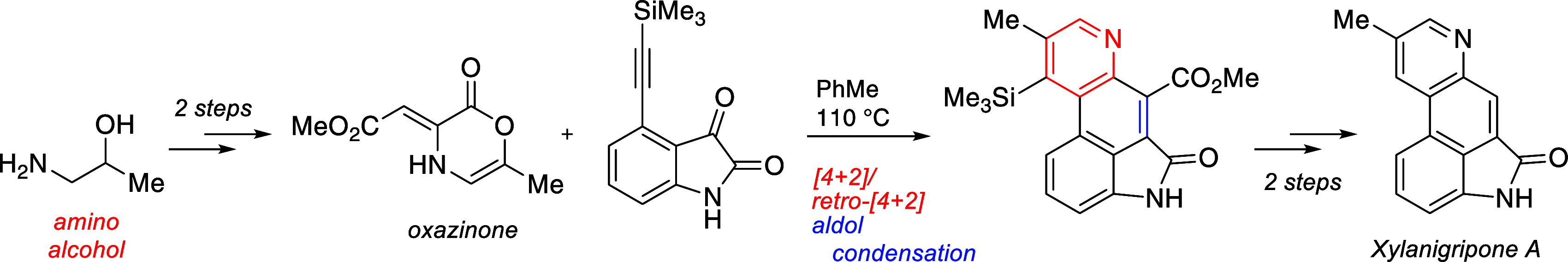

This study describes a general method for the preparation of 1,4-oxazin-2-one intermediates from acetylene dicarboxylate and β-amino alcohol precursors. Oxazinones prepared in this manner were employed in a tandem cycloaddition/cycloreversion reaction sequence with a model alkyne (phenyl acetylene) to give substituted pyridine products. Fundamental reactivity and selectivity studies are complemented by the synthesis of the polycyclic ergot alkaloid natural product xylanigripone A.

## Introduction

The pyridine ring is a privileged heterocyclic scaffold in medicinal chemistry and is frequently present in therapeutic bioactive molecules, agrochemicals, and natural products.^1,2^ Recent technologies focused on C–H activation and functionalization of pyridines and related derivatives (such as pyridinium ions) through both ionic and radical (*e.g*., Minisci-like) processes have enabled several distinct strategies for derivatization of the pyridine nucleus.^3−13^ The preparation of highly substituted pyridine motifs either by classic condensation strategies, modern *de novo* methods of construction, or by C–H activation of intact pyridines remains a challenge. Highly substituted pyridines, such as 2,6-disubstituted variants, can fail in the pyridinium formation or other N-activation step. Merged cycloaddition/cycloreversion processes have proven a reliable method for the preparation of highly substituted aromatic heterocycles and carbocycles.^14−18^

**Figure 1:**
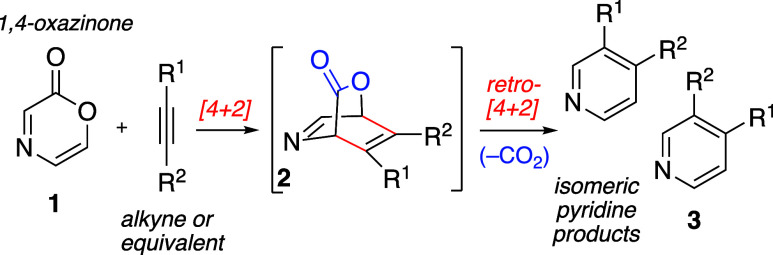
Diels–Alder/retro Diels–Alder sequence with 1,4-oxazinone.

1,4-Oxazinone precursors resembling **1** are the most reactive substrates that can undergo cycloaddition/cycloreversion sequences leading to pyridines (Figure 1). This pericyclic reaction subgroup includes 1,4-oxazinones, 1,2,4-, and 1,2,3-triazines, as well as diazines and other heterocyclic precursors. Computational predictions suggest that **1** has cycloaddition activation energies approximately 4 kcal/mol lower than the complementary 1,2,4-triazine, a more commonly used reactive precursor for the synthesis of pyridines by merged cycloaddition/cycloreversion sequences.^19^ The increased reactivity of oxazinones allow reaction under both normal- and inverse-electron-demand conditions and **1** is competent with a wide array of 2π reaction components, including unactivated alkynes.^20−22^ Cycloaddition with alkynes gives the resulting intermediate adduct represented by **2** (or its regioisomeric complement). The intermediate [2.2.2]bicycloalkene is generally not stable even at ambient temperatures and undergoes extrusion of CO_2_ to afford pyridine products such as **3**.^23^

Our lab is interested in advancing new methods for the construction of oxazinone precursors and exploring their reactivity in the preparation of complex pyridines. This manuscript aligns with this focus and describes a general method for the synthesis of oxazinones from β-amino alcohols and acetylene dicarboxylate. The reactivity of the oxazinones thus produced, as well as the isomeric selectivity in the pyridines obtained through a merged [4 + 2]/retro[4 + 2], is also explored in this report.

**Figure 2:**
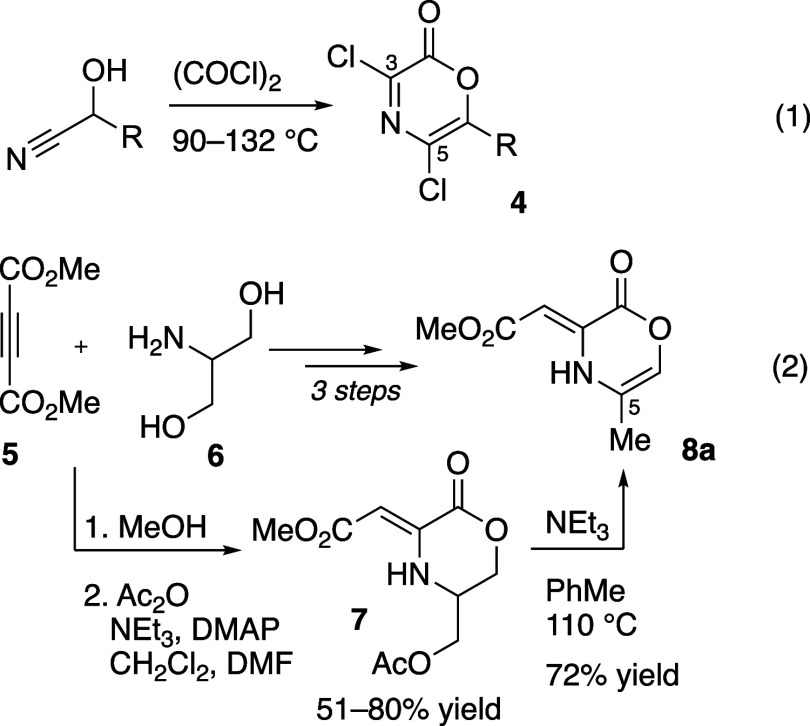
Original and recent method preparing oxazinone intermediates.

The preparation of oxazinone precursors has largely followed the original conditions reported by Hoornaert and co-workers where cyanohydrin derivatives are consumed with excess oxalyl chloride at elevated temperatures (>90 °C) leading to the 3,5-dichloro substituted oxazinones represented by **4** (Figure 2, eq 1).^24,25^ More recently, other conditions have been reported for the preparation of oxazinones bearing other substitution patterns, in particular, those bearing alkyl and aryl groups at positions 3 and 5.^26^ We reported a route to oxazinone **8a** from acetylene dicarboxylate (DMAD, **5**) and aminopropane-1,3-diol **6** (Figure 2, eq 2).^27^ For this particular oxazinone (**8a**), heteroconjugate addition, lactonization, and acylation gives an intermediate dihydrooxazinone **7** (in 51% or 80% yield following recrystallization or chromatography). Elimination of the acetate in **7** with NEt_3_ in toluene at 110 °C and concomitant isomerization gave the 5-methyloxazinone **8a**, which exists in the exocyclic vinylogous urethane tautomer. Oxazinone **8a** has proven competent in the cycloaddition/cycloreversion sequence with several alkynes.^27,28^

## Results and Discussion

Although the sequence from DMAD (**5**) to 5-methyloxazinone **8a** was effective, the nature of the synthesis was specific. We wish to report a complementary and more general sequence to prepare Diels–Alder reactive oxazinones from a variety of β-amino alcohols and acetylene dicarboxylate precursors. Using the chemistry highlighted in Table 1, eight oxazinone intermediates were prepared which feature different groups at position 5 (**8a**–**8d**, entries 1–4,), position 6 (**8e** and **8f**, entries 5, 6) and the two 5,6-disubsituted variants **8g** and **8h** (entries 7, 8). The route toward oxazinones **8a**–**8e** begins with heteroconjugate addition of the β-amino alcohol substrate. The resulting addition product (not shown) undergoes lactonization to the dihydrooxazinone represented by structure **9**. In accord with gearing effects analogous to those described by Thorpe and Ingold,^29^ amino alcohol substrates with substitution proximal to the amino function (R^1^ = C) underwent spontaneous lactonization to **9** at ambient temperatures; substrates lacking substitution or bearing a smaller group at position 5 (entries 5, 6, 8) required heating in methanol (66 °C) to promote lactone formation.

**Table 1:**
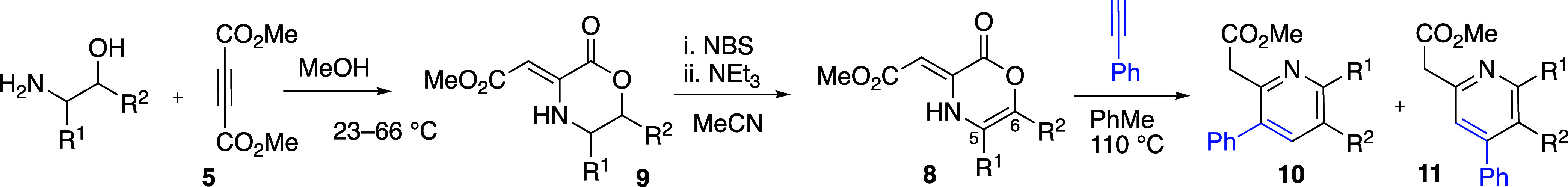

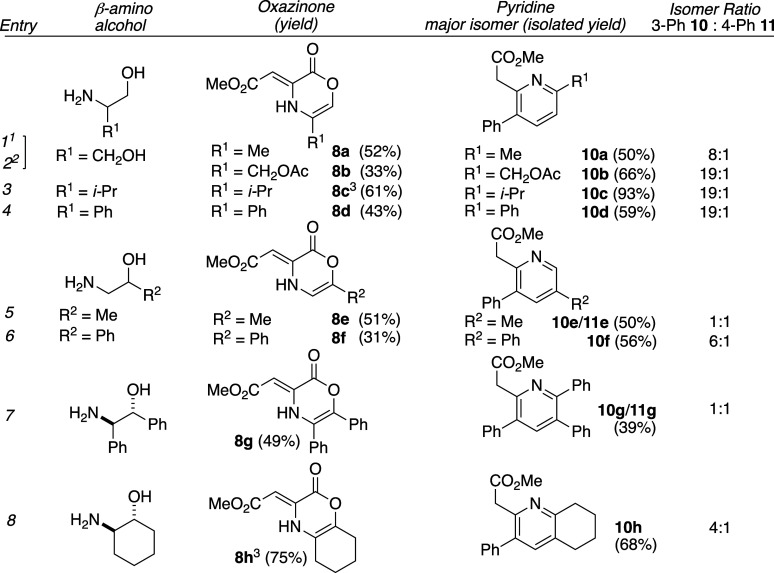
Preparation of Oxazinone Precursors and Cycloaddition/cycloreversion to Afford Pyridine Products

a Key: Oxazinone **8a** prepared through a modified sequence. See experimental information for details.

b Acylation was performed on the intermediate dihydrooxazinone **9** (where R^1^ = CH_2_OH to CH_2_OAc)

c DBU was used for dehydrobromination to give **8c** and **8h**.

Although several net oxidations were considered, we found that a bromination/dehydrobromination sequence was reliable for the conversion of dihydrooxazinone precursors (**9**) into the Diels–Alder-reactive substrates represented by **8**. This was accomplished by exposing **9** to NBS in MeCN, followed by elimination using NEt_3_. All oxazinones were prepared in this manner excluding substrates **8c** and **8h**, where a stronger organic base (DBU) was employed for the dehydrobromination step.

The merged cycloaddition/cycloreversion of oxazinones **8a**–**8h** was explored using a model alkyne, phenylacetylene (Table 1). Efficient reaction was observed in all cases and, overall, 5-substituted oxazinones provided the resulting pyridines **10a**–**10d** in excellent isomeric purity; little to none of the 4-phenyl pyridine isomer **11** was observed in these cases. Reaction with either the 6-substituted oxazinones (**8e** and **8f**) or oxazinones bearing 5,6-disubstitution (**8g** and **8h**) showed lower regioselectivity in the cycloaddition event and the derived pyridine products were afforded as isomeric mixtures ranging from 6:1 to 1:1.

**Scheme 1:**
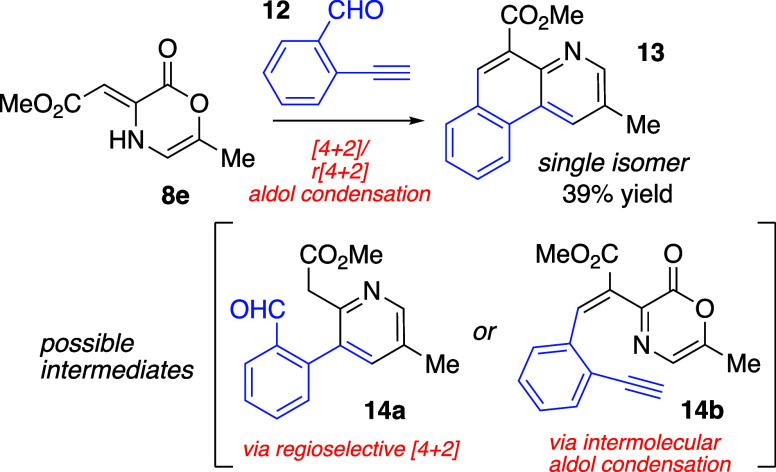
Merged Cycloaddition, Cycloreversion, and Aldol Condensation with 2-ethynylbenzaldehyde

Although the 6-methyloxazinone **8e** was unselective with phenyl acetylene and gave the resulting pyridines **10e** and **11e** in a 1:1 ratio, the merged cycloaddition/cycloreversion sequence with 2-ethynyl benzaldehyde (**12**) gave a single isomer of the fused tricyclic pyridine **13** (Scheme 1). This reaction was very clean, and no other products were observed in the unpurified reaction mixture; only a small amount of unreacted starting material was present. Pyridine **13** can arise from a domino reaction sequence comprised of cycloaddition, extrusion of CO_2_ (cycloreversion), and aldol condensation. Either intermediate **14a** or **14b** could be operational in the regioselective synthesis of **13**. We were not certain whether the pericyclic processes precede an intramolecular aldol condensation, or the alternate scenario, where the aldol condensation ensues prior to an intramolecular cycloaddition. The exceptional improvement in regioselection as compared to phenylacetylene possibly supports the later mechanistic sequence and interception of the intermediate **14b**.

**Scheme 2:**
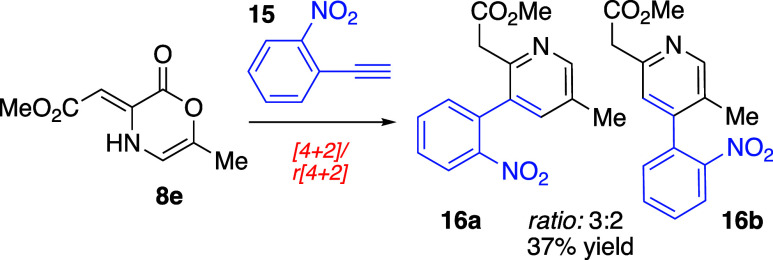
Cycloaddition and Cycloreversion with 2-Nitrophenylacetylene

In order to gain a deeper understanding of the mechanistic sequence of events leading from oxazinone **8e** and 1-ethynylbenzaldehyde (**12**) to the tricycylic product **13**, we performed an analogous sequence using 2-nitrophenylacetylene (**15**) (Scheme 2). Nitrophenylacetylene possesses a similar electronic and steric profile to **12**; however, lacking the 2-carboxaldehyde function, it cannot participate in the aldol condensation. In the event, reaction of oxazinone **8e** and nitrophenylacetylene **15** afforded both pyridine isomeric products **16a** and **16b** in a 3:2 ratio as judged by ^1^H NMR analysis of the unpurified reaction mixture. The structure of the major isomer **16a** was confirmed by NOE. This experiment demonstrates that incorporation of an electron withdrawing group on phenyl acetylene (such as the nitro group in **15**) has only a modest impact on the cycloaddition regioselectivity. This unexceptional selectivity increase (from 1:1 with phenyl acetylene to 3:2 with nitrophenylacetylene) suggests that the reaction between **8e** and 1-ethynylbenzaldehyde (**12**) likely proceeds by aldol condensation prior to pericyclic operations.

**Scheme 3:**
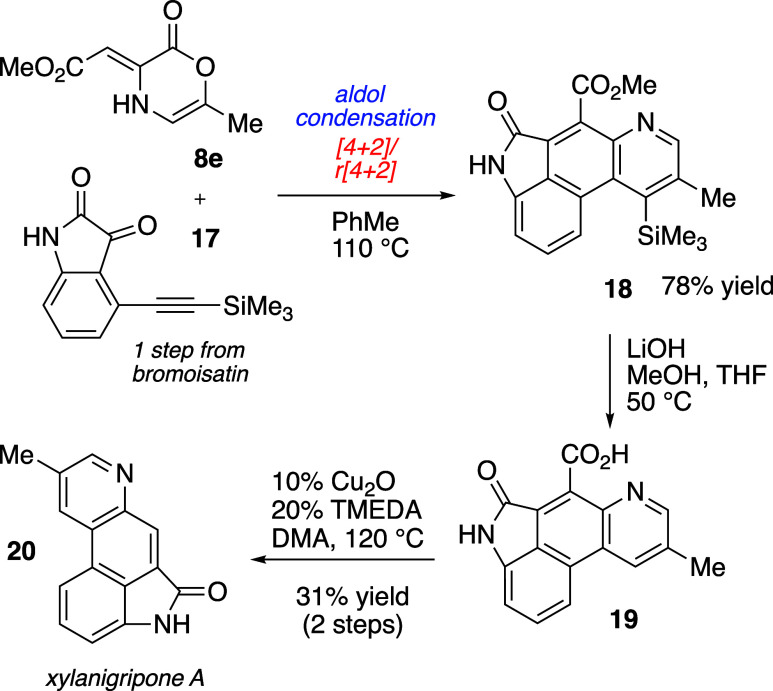
Synthesis of Xylanigripone A

We recognized that an analogous domino reaction sequence might enable access to the tetracyclic pyridine scaffold present in xylanigripone A (**20**), an unusual ergot alkaloid isolated from the rare *Xylaria nigripes* fungi present in abandoned termite nests (Scheme 3).^30^ Historically, *Xylaria* fungal sources have been used to treat diseases of the digestive and central nervous systems.^31−33^ The xylanigripones and related derivatives could serve as important scaffolds for development; however, these compounds are obtained in small quantities from the fungi. Since isolation of the xylanigripones in 2017, two syntheses of xylanigripone A have been disclosed.^34,35^

Our synthetic strategy toward xylanigripone A (**20**) initiated from 6-methyloxazinone **8e**. Union of this starting material with the derived alkynyl isatin **17** led, following aldol condensation and [4 + 2]/retro[4 + 2], to the fused pyridine product **18** as a single isomer in 78% yield. Concomitant cleavage of the methyl ester and silyl function in **18** was achieved with LiOH in MeOH/THF at 50 °C to give penultimate intermediate **19**. Intermediate **19** was challenging to manipulate due to the presence of both pyridine and carboxylic acid moieties. Compound **19** was precipitated directly from the hydrolysis reaction and used without purification in the final operation, a copper-catalyzed decarboxylation, to deliver the natural product xylanigripone A (**20**) in 31% yield from **18** (2 steps).^36^ Overall, the synthetic route to **20** was accomplished in 6 total steps from common reagents (5 steps by the longest linear route).

In conclusion, this report advances a general strategy for the construction of substituted oxazinone precursors from readily available starting materials, β-amino alcohols and acetylene dicarboxylate. Oxazinones prepared by this method were explored in merged cycloaddition/cycloreversion reaction sequences with alkyne reaction components. This chemistry adds to our current knowledge of the reactivity and selectivity of oxazinone intermediates and increases the synthetic utility of these precursors for complex pyridine synthesis. The “one pot” selective construction of the fused polycyclic pyridine products by merging an aldol condensation with the pericyclic processes enabled the efficient construction of the ergot alkaloid skeleton present in xylanigripone A.

## Experimental Section

### General Experimental Considerations

All reactions were carried out under an atmosphere of nitrogen in flame-dried or oven-dried glassware with magnetic stirring or in vials sealed with a Teflon cap. Reagents were used as received. Flash column chromatography was performed using P60 silica gel (230–400 mesh). Analytical thin layer chromatography (TLC) was performed on SiliCycle 60 Å glass plates. Visualization was accomplished with UV light, ceric ammonium molybdate or potassium permanganate, followed by heating. Infrared spectra were recorded using a Digilab FTS 7000 FTIR spectrophotometer. ^1^H NMR spectra were recorded on a 400 MHz spectrometer and are reported in ppm using solvent as an internal standard (CHCl_3_ at 7.26 ppm) or tetramethylsilane (0.00 ppm). Proton-decoupled ^13^C NMR spectra (^13^C{^1^H} NMR) were recorded with a 100 MHz spectrometer and are reported in ppm using solvent as an internal standard (CHCl_3_ at 77.0 ppm, DMSO at 39.5 ppm, pyridine at 150.29 ppm). All compounds were judged to be homogeneous (>95% purity) by ^1^H and ^13^C NMR spectroscopy unless otherwise noted. Mass spectra data analysis was obtained through positive electrospray ionization (w/NaCl) on a Bruker 12 T APEX–Qe FTICR-MS with an Apollo II ion source using an ICR (ion cyclotron resonance) ion trap mass analyzer.

#### General Procedure A

Preparation of 1,4-Oxazinone **8**

A dry flask was charged with a 1,2-amino alcohol (1 equiv) and dissolved in MeOH (0.2 M). The flask was flushed with N_2_ (10 min) and DMAD (1 equiv) was added dropwise. The reaction was allowed to stir at rt until the addition and lactone formation was complete as judged by TLC (1 h). If lactone formation was not complete after 1 h, the reaction mixture was fitted with a condenser and heated to reflux using an aluminum heating block. Following lactone formation, the reaction mixture was concentrated *in vacuo* and the residue was dissolved in MeCN (0.2 M) and flushed with N_2_ (10 min). The reaction was cooled to 0 °C and NBS (1 equiv) was added. After stirring for 5 min at 0 °C, NEt_3_ (2 equiv) was introduced, and the reaction was stirred at rt until dehydrobromination was complete (0.5–16 h) as judged by TLC. The reaction mixture was transferred to separatory funnel and partitioned between 0.1 M HCl and Et_2_O. The organic portion was removed, and the aqueous portion was extracted with additional Et_2_O. The combined organic portions were washed with brine, dried (Na_2_SO_4_), filtered, and concentrated *in vacuo*. The resulting residue was purified by flash column chromatography on silica gel (gradient elution: EtOAc/hexane) to afford the desired 1,4-oxazinone products **8**.

The experimental reaction conditions and data for oxazinone **8a** and pyridine **10a** are reported elsewhere.^27^ Conditions and data for all new compounds are disclosed below.
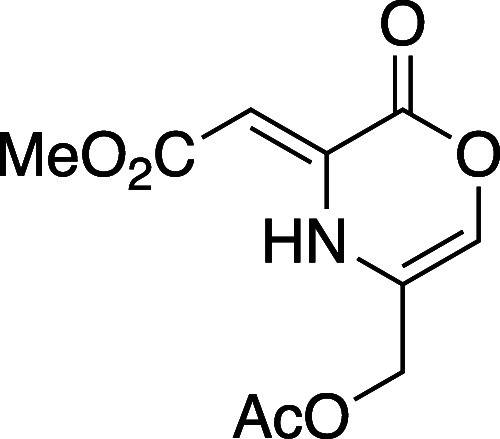


#### Methyl(*Z*)-2-(5-(acetoxymethyl)-2-oxo-2*H*-1,4-oxazin-3(4*H*)-ylidene)acetate (**8b**)

This compound was prepared using a modified version of general procedure A: A dry flask was charged with dihydrooxazinone precursor **7**^27^ (1.0 g, 4.11 mmol) and dissolved in MeCN (21 mL, 0.2M). The flask was flushed with N_2_ for 10 min and cooled to 0 °C. NBS (0.73 g, 4.11 mmol) was added in one portion. The reaction mixture was then stirred for 15 min at 0 °C, until bromination was complete as judged by TLC. NEt_3_ (1.15 mL, 8.22 mmol) was then added dropwise, and the reaction was stirred for 15 min until elimination was complete as judged by TLC. The resulting mixture was transferred to a separatory funnel and partitioned between 0.5 M HCl (10 mL) and Et_2_O (10 mL). The organic layer was removed, and the aqueous layer was extracted with additional Et_2_O (3 × 10 mL). The combined organic layers were washed with brine (10 mL), dried (Na_2_SO_4_), filtered, and concentrated *in vacuo*. The resulting residue was purified by flash column chromatography on silica gel (gradient elution: 10 to 80% EtOAc in hexane) to afford oxazinone **8b** as a light-yellow amorphous solid (320 mg, 33% yield). TLC (20% EtOAc), *R*_f_: 0.25 (CAM); IR (film) 3123, 1744, 1630, 1429, 1262, 764 cm^–1^; ^1^H NMR (400 MHz, CDCl_3_) 10.29 (s, 1H), 6.49 (d, *J* = 2.4 Hz, 1H), 5.85 (d, *J* = 0.8 Hz, 1H), 4.68 (s, 2H), 3.77 (s, 3H), 2.15 (s, 3H); ^13^C{^1^H} NMR (100 MHz, CDCl_3_) δ 171.1, 169.7, 156.1, 138.3, 125.0, 118.3, 90.0, 59.3, 51.3, 20.5; HRMS (ESI) *m*/*z*: [M + Na]^+^ Calcd for C_10_H_11_NO_6_Na^+^ 264.0478; Found 264.0477.
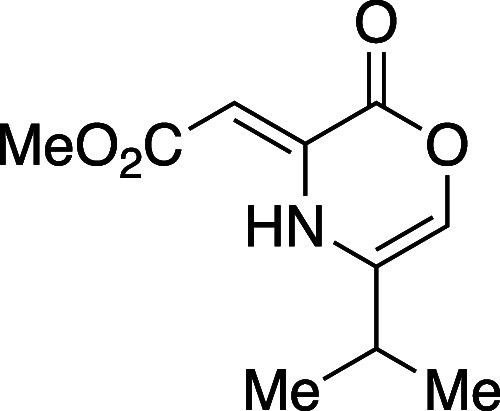


#### Methyl (*Z*)-2-(5-Isopropyl-2-oxo-2*H*-1,4-oxazin-3(4*H*)-ylidene)acetate (**8c**)

Oxazinone **8c** was synthesized following a modified general procedure A, using DBU in the place of NEt_3_, to afford an amorphous yellow solid (980 mg, 61% yield). TLC (10% EtOAc), *R*_f_: 0.60 (CAM); IR (film) 1745, 1653, 1250, 1159, 1031, 626 cm^–1^; ^1^H NMR (400 MHz, CDCl_3_) 10.22 (s, 1H), 6.27 (d, *J* = 2.0 Hz, 1H), 5.80 (s, 1H), 3.76 (s, 3H), 2.52 (sept, *J* = 7.2 Hz, 1H), 1.22 (d, *J* = 6.8 Hz, 6H); ^13^C{^1^H} NMR (100 MHz, CDCl_3_) δ 170.7, 157.0, 139.6, 127.4, 121.1, 88.2, 51.3, 27.9, 20.3; HRMS (ESI) *m*/*z*: [M + Na]^+^ Calcd for C_10_H_13_NO_4_Na^+^ 234.0737; Found 234.0738.
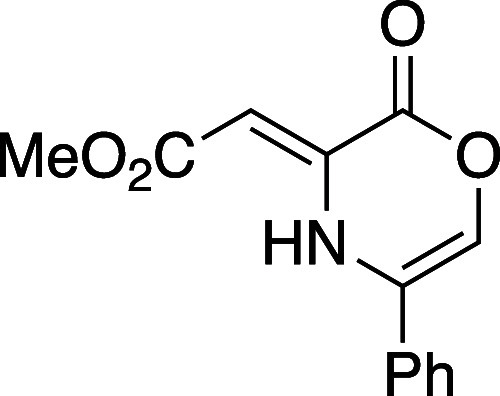


#### Methyl (*Z*)-2-(2-Oxo-5-phenyl-2*H*-1,4-oxazin-3(4*H*)-ylidene)acetate (**8d**)

Following general procedure A, oxazinone **8d** was prepared as a yellow powder (54 mg, 43% yield). Mp 136–142 °C; TLC (20% EtOAc), *R*_f_: 0.5 (CAM); IR (film) 3941, 3252, 3130 3005, 2955, 2845, 1794, 1661, 1614, 1435, 1282, 1261, 1198, 1180, 1147, 1030, 1015, 760 cm^–1^; ^1^H NMR (400 MHz, CDCl_3_) 10.65 (s, 1H), 7.47–7.34 (m, 5H), 6.73 (d, *J* = 2.8 Hz, 1H), 5.91 (s, 1H), 3.78 (s, 3H); ^13^C{^1^H} NMR (100 MHz, CDCl_3_) δ 170.6, 156.5, 139.0, 130.0, 129.6, 129.4, 124.7, 123.0, 122.5, 89.4, 51.4; HRMS (ESI) *m*/*z*: [M + Na]^+^ Calcd for C_13_H_11_NO_4_Na^+^ 268.0580; Found 268.0581.
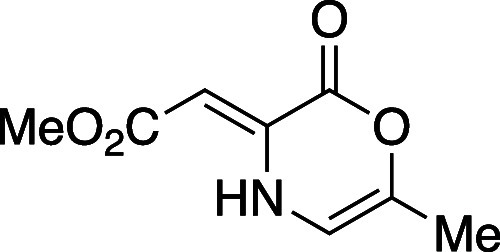


#### Methyl (*Z*)-2-(6-Methyl-2-oxo-2*H*-1,4-oxazin-3(4*H*)-ylidene)acetate (**8e**)

Following general procedure A, oxazinone **8e** was prepared as an amorphous yellow solid (790 mg, 51% yield). TLC (60% EtOAc in hexane), *R*_f_: 0.95 (CAM); IR (film) 1744, 1651, 1601, 1435, 1269, 1138, 1084, 764 cm^–1^; ^1^H NMR (400 MHz, CDCl_3_) 10.03 (s, 1H), 5.99 (d, *J* = 2.8 Hz, 1H), 5.76 (s, 1H), 3.73 (s, 3H), 2.01 (d, *J* = 1.2 Hz, 3H); ^13^C{^1^H} NMR (100 MHz, CDCl_3_) δ 170.6, 157.4, 138.6, 134.8, 105.0, 87.5, 51.2, 16.1; HRMS (ESI) *m*/*z*: [M + Na]^+^ Calcd for C_8_H_9_NO_4_Na^+^ 206.0424; Found 206.0424.
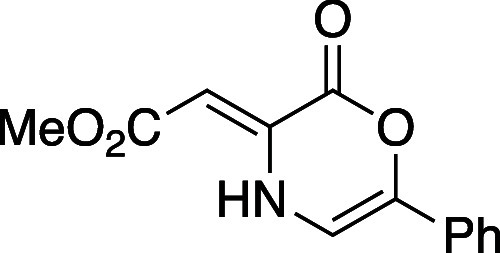


#### (*Z*)-2-Ethylidene-5-Phenyl-1,4-dihydropyridin-3(2*H*)-one--carbon dioxide (**8f**)

Oxazinone **8f** was synthesized following a modified general procedure A. After 24 h of heating in MeOH to promote lactonization, a portion of unlactonized material remained and was removed through chromatographic separation (gradient elution: 7–100% EtOAc in hexanes) before proceeding with the bromination/elimination sequence. Oxazinone **8f** was obtained as a yellow powder (390 mg, 31% yield). Mp: 160–166 °C; TLC (20% EtOAc in hexane), *R*_f_: 0.40 (CAM); IR (film) 1761, 1748, 1593, 1435, 1146, 758 cm^–1^; ^1^H NMR (400 MHz, CDCl_3_): 10.39 (s, 1H), 7.54 (d, *J* = 8.8 Hz, 2H), 7.41–7.29 (m, 4H), 6.74 (d, *J* = 5.6 Hz, 1H), 5.89 (s, 1H), 3.77 (s, 3H); ^13^C{^1^H} NMR (100 MHz, CDCl_3_) δ 170.4, 156.7, 138.1, 136.4, 130.4, 129.2, 128.8, 128.3, 122.7, 105.1, 89.2, 51.4; HRMS (ESI) *m*/*z*: [M + Na]^+^ Calcd for C_13_H_11_NO_4_Na^+^ 268.0580; Found 268.0580.
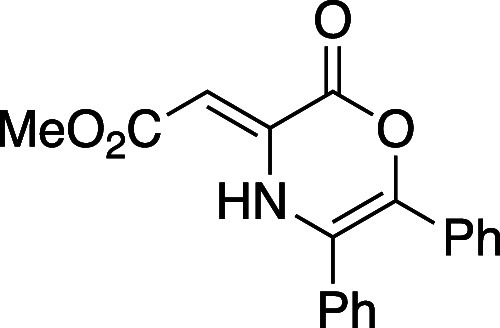


#### Methyl (*Z*)-2-(2-Oxo-5,6-diphenyl-2*H*-1,4-oxazin-3(4*H*)-ylidene)acetate (**8g**)

Following general procedure A, oxazinone **8g** was prepared as an amorphous yellow solid (490 mg, 49% yield). TLC (10% EtOAc), *R*_f_: 0.60 (CAM); IR (film) 1755, 1622, 1312, 1275, 1252, 694 cm^–1^; ^1^H NMR (400 MHz, CDCl_3_) 10.44 (s, 1H), 7.39 (s, 5H), 7.21 (s, 5H), 5.90 (s, 1H), 3.75 (s, 3H) ^13^C{^1^H} NMR (100 MHz, CDCl_3_) δ 1.1, 186.5, 170.6, 156.8, 138.4, 133.3, 132.2, 131.2, 129.6, 129.4, 128.8, 128.3, 128.1, 127.9, 119.6, 88.5, 51.4; HRMS (ESI) *m*/*z*: [M + Na]^+^ Calcd for C_19_H_15_NO_4_Na^+^ 344.0893; Found 344.0895.
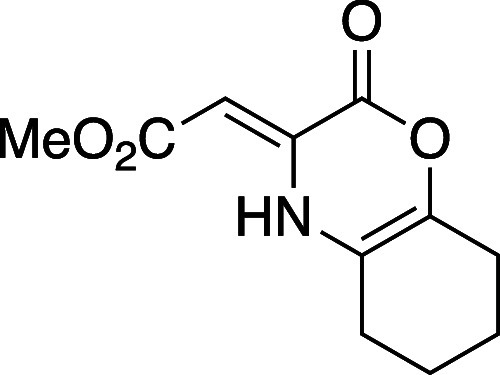


#### Methyl (*Z*)-2-(2-Oxo-5,6,7,8-tetrahydro-2*H*-benzo[*b*][1,4]oxazin-3(4*H*)-ylidene)acetate (**8h**)

Oxazinone **8h** was synthesized following a modified general procedure A, using DBU in the place of NEt_3_, to afford an amorphous yellow solid (57 mg, 75% yield). TLC (20% EtOAc), *R*_f_: 0.25 (CAM); IR (film) 1748, 1620, 1341, 1136, 768 cm^–1^; ^1^H NMR (400 MHz, CDCl_3_) 9.90 (s, 1H), 5.74 (s, 1H), 3.74 (s, 3H), 2.32 (m, 4H), 1.75 (m, 4H); ^13^C{^1^H} NMR (100 MHz, CDCl_3_) δ 170.7, 157.6, 139.7, 132.3, 114.5, 87.9, 51.1, 24.8, 24.4, 21.8, 21.6; HRMS (ESI) *m*/*z*: [M + Na]^+^ Calcd for C_11_H_13_NO_4_Na^+^ 246.0736; Found 246.0737.

#### General Procedure B

Synthesis of Pyridine Isomers **10** and/or **11** through [4 + 2]/retro[4 + 2].

A dry flask was charged with oxazinone **8** and dissolved in a 1:1 mixture of PhMe and phenylacetylene (0.5M). The vial was flushed with N_2_ (5 min), sealed with a Teflon cap, and heated to 110 °C overnight using an aluminum heating block. After cooling rt and concentration *in vacuo*, the resulting unpurified reaction mixture was analyzed by ^1^H NMR to determine the isomeric ratio of pyridines **10** and **11**. The mixture was purified by flash column chromatography on silica gel (gradient elution: EtOAc/hexane).
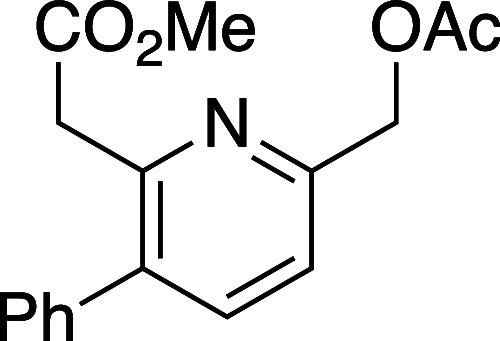


#### Methyl 2-(6-(Acetoxymethyl)-3-phenylpyridin-2-yl)acetate (**10b**)

Following general procedure B, pyridine **10b** was synthesized as a light-yellow oil (61 mg, 66% yield). TLC (30% EtOAc in hexane), *R*_f_: 0.68 (CAM); IR film 3059, 2951, 1736, 1591, 1435, 1007, 704 cm^–1^; ^1^H NMR (400 MHz, CDCl_3_) 7.60 (d, *J* = 7.6 Hz, 1H), 7.44–7.30 (m, 6H), 5.26 (s, 2H), 3.84 (s, 2H), 3.64 (s, 3H), 2.18 (s, 3H); ^13^C{^1^H} NMR (100 MHz, CDCl_3_) 171.2, 170.6, 154.4, 151.8, 138.7, 138.4, 136.9, 128.9, 128.5, 127.9, 120.2, 66.7, 51.9, 41.6, 20.9; HRMS (ESI) *m*/*z*: [M + H]^+^ Calcd for C_17_H_17_NO_4_H^+^ 300.1230; Found 300.1229.
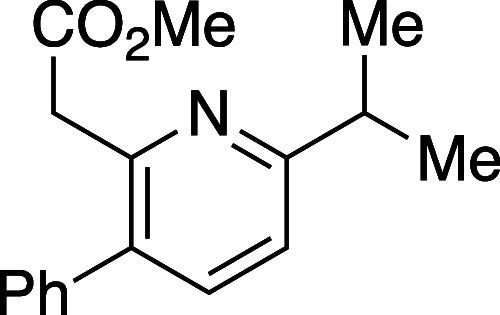


#### Methyl 2-(6-Isopropyl-3-phenylpyridin-2-yl)acetate (**10c**)

Following general procedure B, pyridine **10c** was synthesized as a light-yellow oil (48 mg, 93% yield). TLC (10% EtOAc in hexane), *R*_f_: 0.60 (CAM); IR film 1740, 1591, 1436, 1158, 1007, 726 cm^–1^; ^1^H NMR (400 MHz, CDCl_3_) 7.51 (d, *J* = 7.6 Hz, 1H), 7.41 (m, 3H), 7.33 (m, 2H), 7.16 (d, *J* = 8.4 Hz, 1H), 3.82 (s, 2H), 3.64 (s, 3H), 3.09 (m, 1H), 1.33 (d, *J =* 7.2 Hz, 6H); ^13^C{^1^H} NMR (100 MHz, CDCl_3_) δ 171.6, 166.0, 151.0, 139.4, 138.0, 134.8, 129.0, 128.4, 127.5, 118.6, 51.8, 41.9, 36.0, 22.6; HRMS (ESI) *m*/*z*: [M + Na]^+^ Calcd for C_17_H_19_NO_2_Na^+^ 292.1308; Found: 292.1309.
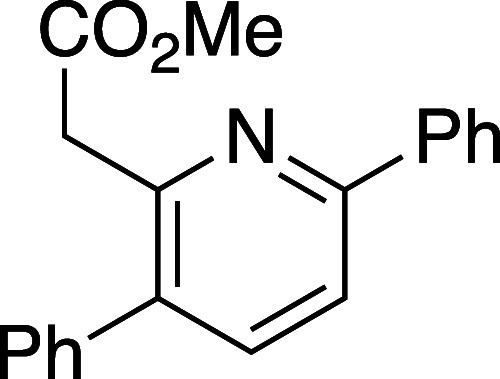


#### Methyl 2-(3,6-Diphenylpyridin-2-yl)acetate (**10d**)

Following general procedure B, pyridine **10b** was synthesized as a light-yellow oil (69 mg, 59% yield). TLC (30% EtOAc in hexane), *R*_f_: 0.25 (CAM); IR film 1734, 1456, 1433, 1337, 1230, 1200, 1163, 1153, 1007, 708 cm^–1^; ^1^H NMR (400 MHz, CDCl_3_) 8.06 (s, 1H), 7.72, (d, *J* = 8.4 Hz, 1H), 7.66 (d, *J* = 8.4 Hz, 1H), 7.48–7.36 (m, 10H), 3.93 (s, 2H), 3.67 (s, 3H); ^13^C{^1^H} NMR (100 MHz, CDCl_3_) δ 171.6, 155.8, 151.9, 139.1, 138.9, 138.4, 134.0, 129.0, 128.9, 128.7, 128.6, 127.8, 126.9, 118.7, 52.0, 42.1; HRMS (ESI) *m*/*z*: [M + H]^+^ Calcd for C_20_H_18_NO_2_^+^ 304.1332; Found 304.1331.
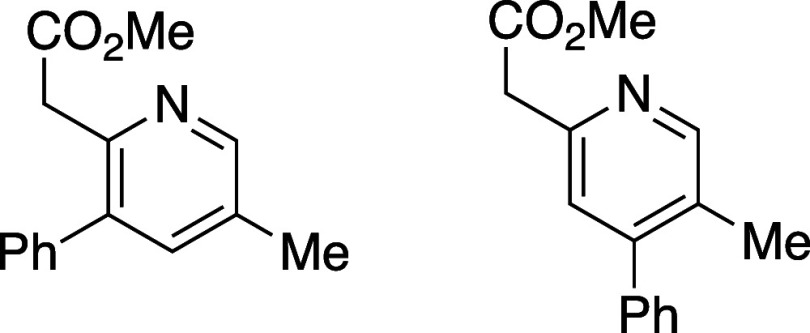


#### Methyl 2-(5-Methyl-3-phenylpyridin-2-yl)acetate and Methyl 2-(5-methyl-4-phenylpyridin-2-yl)acetate (**10**e**/11e**)

Following general procedure B, pyridines **10e** and **11e** were synthesized as a light-yellow oil (18 mg, 50% yield). The resulting isomers were inseparable by flash column chromatography and are characterized as a mixture. TLC (10% EtOAc in hexane), *R*_f_: 0.60 (CAM); IR film 1736, 1435, 1335, 1161, 1007, 775 cm^–1^; ^1^H NMR (400 MHz, CDCl_3_) 8.45 (s, 1H), 8.42 (s, 1H), 7.45–7.32 (m, 5H), 7.38–7.26 (m, 5H), 3.86 (s, 2H), 3.80 (s, 2H), 2.36 (s, 3H), 2.26 (s, 3H);^13^C{^1^H} NMR (100 MHz, CDCl_3_) δ 171.6, 171.2, 151.7, 150.9, 149.9, 149.1, 148.9, 130.1, 138.92, 138.4, 137.2, 131.6, 129.2, 128.9, 128.5, 128.5, 128.4, 128.0, 127.7, 124.1, 52. 2, 52.0, 43.2, 41.1, 18.0, 16.9; HRMS (ESI) *m*/*z*: [M + H]^+^ Calcd for C_15_H_15_NO_2_H^+^ 242.1175; Found 242.1174.
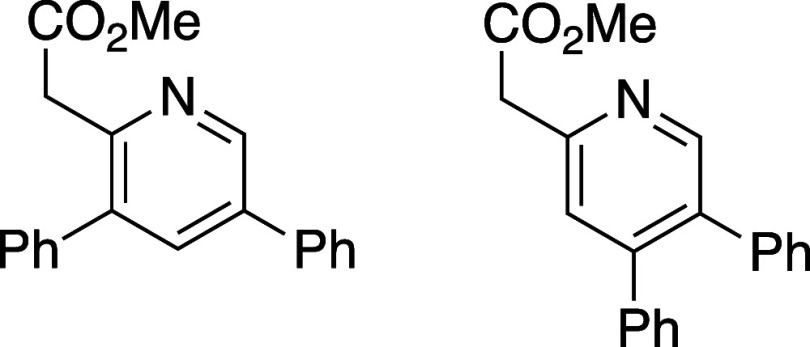


#### Methyl 2-(3,5-Diphenylpyridin-2-yl)acetate (**10***f***/11f**)

Following general procedure B, pyridines **10f** and **11f** were synthesized as a light-yellow oil (35 mg, 56% yield). The resulting isomers were inseparable by flash column chromatography and are characterized as a mixture. TLC (30% EtOAc in hexane), *R*_f_: 0.25 (CAM); IR film 1732, 1447, 1435, 1240, 1209, 1157, 1043, 764 cm^–1^; ^1^H NMR (400 MHz, CDCl_3_) 8.59 (s, 1H), 7.35 (s, 1H), 7.26 (m, 4H), 7.15 (m, 4H), 3.95 (s, 2H), 3.77 (s, 3H); ^13^C{^1^H} NMR (100 MHz, CDCl_3_) δ 171.1, 153.2, 150.7, 148.4, 138.5, 137.4, 134.4, 129.8, 129.3, 128.2, 128.2, 127.9, 127.3, 124.7, 52.2, 43.4; HRMS (ESI) *m*/*z*: [M + H]^+^ Calcd for C_20_H_18_NO_2_^+^ 304.1332; Found 304.1331.
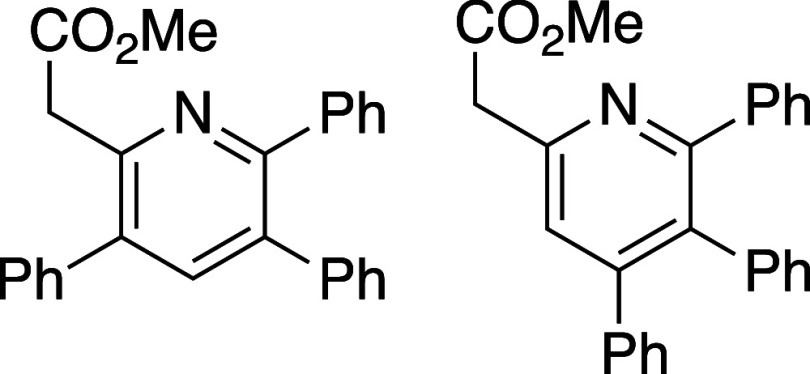


#### Methyl 2-(3,5,6-Triphenylpyridin-2-yl)acetate and methyl 2-(4,5,6-triphenylpyridin-2-yl)acetate (**10***g***/11g**)

Following general procedure B, pyridines **10g** and **11g** were synthesized as a light-yellow oil (27 mg, 39% yield). A small portion of **10g** was obtained for analytical purposes and ^1^H NMR data below is for isomerically pure **10g**; ^13^C{^1^H} NMR data was obtained using a mixture of isomers **10g** and **11g**. TLC (10% EtOAc in hexane), *R*_f_: 0.50 (CAM); IR film 1738, 1537, 1260, 1163, 757 cm^–1^; ^1^H NMR of isomerically pure **10g** (400 MHz, CDCl_3_) 7.35 (s, 1H), 7.25 (m, 3H), 7.19 (m, 5H), 7.06 (m, 5H), 6.87 (m, 2H), 4.01 (s, 2H), 3.77 (s, 3H); ^13^C{^1^H} NMR of isomers **10g** and **11g** (100 MHz, CDCl_3_) δ 171.3, 158.1, 152.8, 150.4, 140.6, 139.3, 137.6, 132.9, 131.4, 130.0, 129.3, 127.8, 127.7, 127.4, 127.3, 126.6, 123.7, 52.1, 43.6; HRMS (ESI) *m*/*z*: [M + Na]^+^ Calcd for C_26_H_21_NO_2_Na^+^ 402.1464; Found 402.1465.
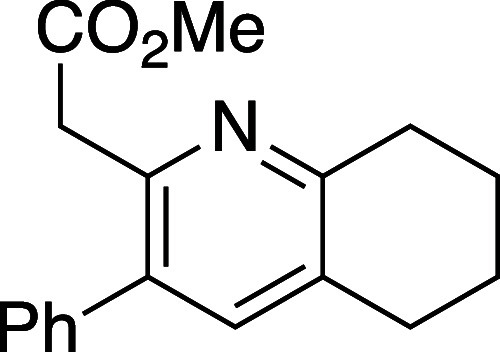


#### Methyl 2-(3-Phenyl-5,6,7,8-tetrahydroquinolin-2-yl)acetate (**10h**)

Following general procedure B, pyridine **10h** was synthesized as a light-yellow oil (19 mg, 68% yield). TLC (20% EtOAc in hexane), *R*_f_: 0.30 (CAM); IR film 1736, 1433, 1155, 702 cm^–1^; ^1^H NMR (400 MHz, CDCl_3_) 7.40–7.28 (m, 5H), 7.25 (s, 1H), 3.78 (s, 2H), 3.63 (s, 3H), 2.95 (t, *J* = 6.4 Hz, 2H), 2.79 (t, *J* = 6.4 Hz, 2H), 1.93–1.91 (m, 2H), 1.85–1.82 (m, 2H); ^13^C{^1^H} NMR (100 MHz, CDCl_3_) δ 171.8, 156.2, 148.7, 139.5, 138.4, 135.0, 130.8, 129.0, 128.4, 127.5, 51.9, 41.4, 32.2, 28.4, 23.1, 22.7; HRMS (ESI) *m*/*z*: [M + Na]^+^ Calcd for C_18_H_19_NO_2_Na^+^ 282.1488; Found 282.1490.
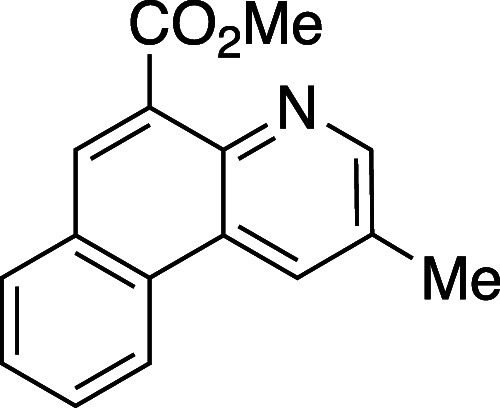


#### Methyl 2-Methylbenzo[*f*]quinoline-5-carboxylate (**13**)

A dry vial was charged with oxazinone **8e** (50 mg, 0.27 mmol, 1 equiv) and 2-ethylyl-benzaldehyde (**12**) (43 mgs, 0.33 mmol, 1.2 equiv), and dissolved in PhMe (1 mL). The resulting solution was heated to 120 °C using an aluminum heating block and stirred for 72 h. The reaction mixture was cooled to rt, concentrated *in vacuo*, and the resulting material was purified by flash column chromatography on silica gel (gradient elution: 0% to 80% EtOAc) to yield pyridine **13** as an amorphous yellow solid (28 mg, 39% yield). TLC: 40% EtOAC in hexanes, R_*f*_. 0.40 (KMnO_4_); IR (film) 1724, 1238, 1209, 1198, 1172, 1024, 808, 752 cm^–1^; ^1^H NMR (400 MHz, CDCl_3_) 8.92 (s, 1H), 8.75 (s, 1H), 8.63 (d, *J* = 8.0 Hz, 1H), 8.28 (s, 1H), 7.98 (d, *J* = 7.6 Hz, 1H), 7.76 (t, *J* = 6.4 Hz, 1H), 7.70 (t, *J* = 6.4 Hz, 1H), 4.09 (s, 3H), 2.63 (s, 3H); ^13^C{^1^H} NMR (100 MHz, CDCl_3_) δ 168.3, 151.5, 143.1, 131.4, 130.5, 130.2, 130.2, 130.1, 129.5, 128.5, 127.6, 125.4, 122.5, 52.6, 19.0; HRMS (ESI) *m*/*z*: [M + H]^+^ Calcd for C_16_H_13_NO_2_H^+^ 252.1019; Found 252.1019.
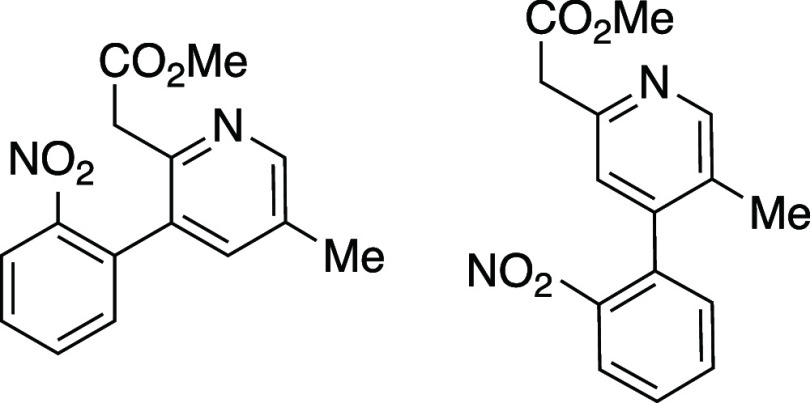


#### Methyl 2-(5-Methyl-3-(2-nitrophenyl)pyridin-2-yl)acetate and Methyl 2-(5-methyl-4-(2-nitrophenyl)pyridin-2-yl)acetate (**16a** and **16b**)

A dry vial was charged with oxazinone **8e** (150 mg, 1 equiv) and 1-ethynyl-2-nitrobenzene (**15**) (142 mg, 1.5 equiv) and dissolved in PhMe (1.5 mL, 0.54M). The resulting solution was heated to 120 °C using an aluminum heating block and stirred for 96 h. The reaction mixture was cooled to rt and concentrated *in vacuo*. ^1^H NMR analysis of the resulting unpurified reaction mixture revealed a 3:2 mixture of pyridine isomers **16a** and **16b**. The resulting material was purified by flask column chromatography on silica gel (gradient elution: 5 to 80% EtOAc in hexanes) to yield **16a** and **16b** as a mixture of isomers as a brown oil (88 mg, 37% yield). The structure of **16a** was confirmed as the major isomer by NOE. The isomers proved inseparable by chromatography and data is reported on a mixture of isomers **16a** and **16b**. TLC (40% EtOAc in hexane), *R*_f_: 0.30 (KMnO_4_); IR (film) 1772, 1734, 1653, 1506, 951 cm^–1^; ^1^H NMR **16a**, major isomer (400 MHz, CDCl_3_) 8.46 (d, *J* = 1.6, 1H), 8.11 (d, *J* = 8.0 Hz, 1H), 7.70 (m, 1H), 7.65 (m, 1H), 7.40 (d, *J* = 6.8, 1H), 7.28 (s, 1H), 3.72 (d, *J* = 14.9, 1H), 3.61 (s, 3H), 3.53 (d, *J* = 16.0, 1H), 2.35 (s, 3H); ^1^H NMR **16b**, minor isomer (400 MHz, CDCl_3_) 8.46 (d, *J* = 1.6, 1H), 8.11 (d, *J* = 7.6, 1H), 7.70 (m, 1H), 7.65 (m, 1H), 7.07 (s, 1H), 3.86 (m, 2H), 3.73 (s, 3H), 2.07 (s, 3H). ^13^C{^1^H} NMR (100 MHz, CDCl_3_) 171.0, 170.9, 151.8, 150.4, 149.7, 149.0, 148.4, 146.6, 137.1, 133.9, 133.5, 133.3, 133.0, 132.9, 132.4, 131.5, 131.3, 129.4, 129.3, 129.3, 124.6, 124.5, 122.7, 52.2, 52.0, 43.2, 41.4, 17.9, 16.3. δ HRMS (ESI) *m*/*z*: [M + Na]^+^ Calcd for C_15_H_14_N_2_O_4_Na^+^ 309.0846; Found 309.0846.
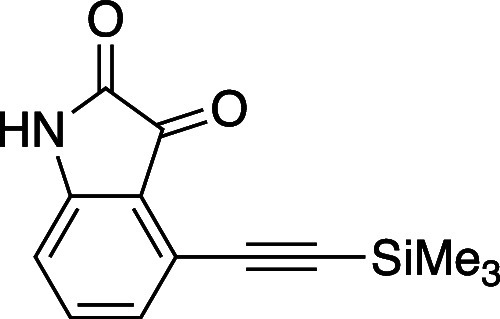


#### 4-[(Trimethylsilyl)ethynyl]-1H-indole-2,3-dione (**17**)

A dry two neck flask was charged with 4-bromoisatin **(**2.00 g, 8.8 mmol) and dissolved in PhMe (56 mL), THF (56 mL), and NEt_3_ (56 mL). The flask was fitted with an air condenser and was evacuated and backfilled with N_2_ gas three times. Sequentially, PdCl_2_(PPh_3_)_2_ (0.92 g, 1.32 mmol), TMS acetylene (1.75 mL, 12.3 mmol), and CuI (166 mg, 0.87 mmol) were added at rt. The reaction mixture was evacuated and backfilled with N_2_ two additional times and the reaction mixture was heated to 50 °C using an aluminum heating block. After heating for 2 h, the reaction was cooled to rt, concentrated *in vacuo*, and partitioned between 20 mL of EtOAc and an equal volume of sat. aqueous NH_4_Cl. The organic layer was removed, and the aqueous layer was extracted with additional EtOAc (3 × 20 mL). The combined organic layers were washed with brine, dried (Na_2_SO_4_), filtered and concentrated *in vacuo.* The resulting residue was purified by flash column chromatography on silica gel (gradient elution: 10% EtOAc to 100% EtOAc in hexanes) to afford **17** (1.64 g, 77% yield) as an amorphous orange solid. Isatin **17** proved sensitive and prone to degradation. TLC (40% EtOAc in hexane), *R*_f_: 0.50 (KMnO_4_); IR (film) 3150, 1736, 1586, 1246, 841 cm^–1^; ^1^H NMR (400 MHz, CDCl_3_) 8.97 (s, 1H), 7.48 (t, *J* = 8.0 Hz, 1H), 7.13 (dd, *J_1_=* 0.8 Hz, *J*_*2*_ = 8.0 Hz, 1H), 6.95 (dd, *J*_*1*_*=* 0.8 Hz, *J*_*2*_ = 8.0 Hz, 1H) 0.31 (s, 9H); ^13^C{^1^H} NMR (100 MHz, CDCl_3_) δ 181.1, 159.4, 149.4, 137.6, 128.0, 121.8, 117.9, 112.5, 104.7, 100.0, −0.4. HRMS (ESI) *m*/*z*: [M + Na]^+^ Calcd for C_13_H_13_NO_2_SiNa^+^ 266.0608; Found 266.0608.
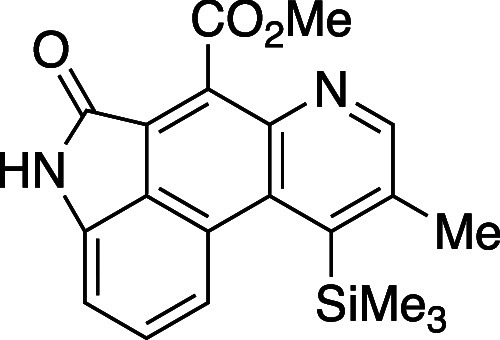


#### Methyl-9-Methyl-5-oxo-10-(trimethylsilyl)-4,5-dihydroindolo[4,3-*fg*]quinoline-6-carboxylate (**18**)

A dry vial was charged with TMS alkyne **17** (20 mg, 0.08 mmol) and oxazinone **8e** (60 mg, 0.08 mmol). The flask was flushed with N_2_ gas for five min and the starting materials dissolved in PhMe (150 μL, 0.5M). The reaction was heated for 16 h at 120 °C using an aluminum heating block. After cooling to rt, the reaction mixture was concentrated *in vacuo* and the resulting residue was purified by flash column chromatography on silica gel (gradient elution: 10% EtOAc in hexanes to 100% EtOAc). The resulting product **18** was obtained as an orange solid (23 mg, 78% yield). Mp: 230–235 °C; TLC (60% EtOAc in hexane), R_*f*_. 0.5 (CAM); IR (film) 1740, 1702, 1653, 1630, 1270, 1226, 1067, 847 cm^–1^; ^**1**^H NMR (400 MHz, CDCl_3_) 8.78 (s, 1H), 7.97 (d, *J =* 8.8 Hz, 1H), 7.69 (s, 1H), 7.55 (m, 1H), 7.07 (d, *J =* 7.2 Hz, 1H), 5.30 (s, 1H), 4.20 (d, *J =* 4.4 Hz, 3H), 2.75 (s, 3H), 0.46 (s, 9H); ^13^C{^1^H} NMR (100 MHz, CDCl_3_) δ 194.9, 167.3, 167.1, 151.4, 146.1, 144.17, 141.6, 137.5, 133.8, 132.8, 127.8, 127.6, 123.4, 122.2, 122.0, 107.9, 53.3, 22.5, 2.8. HRMS (ESI) *m*/*z*: [M + H]^+^ Calcd for C_20_H_20_N_2_O_3_SiH^+^ 365.1316; Found 365.1315.
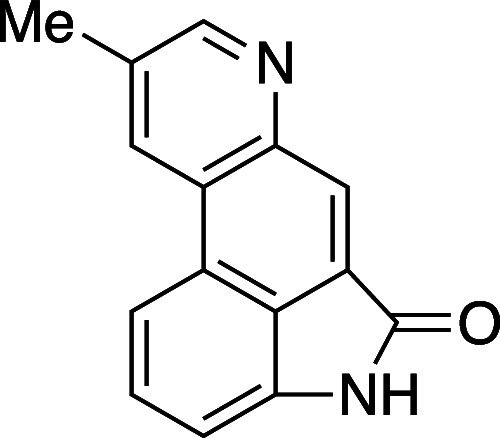


#### 9-Methylindolo[4,3-fg]20uinoline-5(4H)-one (**20**)

A dry vial was charged with **18** (170 mg, 0.467 mmol) and dissolved in MeOH (2 mL) and H_2_O (2 mL). To this solution was added LiOH (98 mg, 2.33 mmol), and the vial was heated to 50 °C for 72 h using an aluminum heating block. The reaction mixture was diluted with 0.1 M HCl (25 mL) and the resulting precipitated solids were removed by vacuum filtration to give carboxylic acid **19** (40 mg, 0.14 mmol) as a red solid, which was used directly in the following reaction without further purification. The material (**19**) thus obtained was transferred to a vial, dissolved in dimethylacetamide (100 μL) and Cu_2_O (0.35 mg, 0.0025 mmol) and TMEDA (1 μL, 0.005 mmol) were added. The vial was sealed with a teflon cap and the reaction vessel was placed in an aluminum heating block set to 140 °C. After heating for 48 h, the reaction mixture was cooled to rt, and concentrated *in vacuo*. The resulting residue was purified via flash column chromatography (gradient elution: 0 to 100% EtOAc in hexanes) to give xylanigripone A (**20**) as a yellow oil (9 mg, 31% yield over two steps from **18**). TLC (60% EtOAc in hexane), R_*f*_. 0.5 (CAM); ^1^H NMR (400 MHz, CDCl_3_): 8.97 (s, 1H), 8.72 (s, 1H), 8.66 (s, 1H), 8.11 (d, *J* = 8.8 Hz, 1H), 7.85 (s, 1H), 7.67 (t, *J* = 8.4 Hz, 1H), 7.13 (d, *J* = 7.2 Hz, 1H), 2.67 (s, 3H). ^1^H NMR (400 MHz, pyridine-*d*_*5*_): 12.38 (s, 1H), 9.00 (s, 1H), 8.96 (s, 1H), 8.80 (s, 1H), 8.25 (d, *J* = 8.4 Hz, 1H), 7.72 (t, *J* = 7.2 Hz, 1H), 2.44 (s, 3H). ^13^C{^1^H} NMR (100 MHz, pyridine-*d*_*5*_): 170.1, 152.8, 148.4, 140.3, 134.0, 131.7, 130.5, 129.7, 128.3, 127.9, 127.3, 116.3, 107.8, 19.1. ^1^H and ^13^C NMR spectroscopic data for **20** are in agreement with published spectra from the isolation work^30^ and prior synthesis efforts.^34,35^

## Data Availability

The data underlying this study are available in the published article and the Supporting Information.

## Supporting Information Available

Supporting Information including ^1^H and ^13^C NMR spectra for all new compounds (PDF)
