# Supplementary material for: Polysubstituted Pyridines from 1,4-Oxazinone Precursors
Source: J Org Chem. 2024 Nov 12;89(23):17635–42. doi: 10.1021/acs.joc.4c02389 (PMC11629385; doi:10.1021/acs.joc.4c02389)
Supplement: Supplementary file 1 — jo4c02389_si_001.pdf [file jo4c02389_si_001.pdf]

Supporting Information

Polysubstituted Pyridines from 1,4-Oxazinone Precursors

L.C. Thompson, Adrianne M. Kinsey, Zannatul Shahla, Jonathan R. Scheerer*

Department of Chemistry, The College of William & Mary, P.O. Box 8795, Williamsburg, Virginia, 23187.

Supporting Information

^{1}H and ^{13}C NMR Spectra S2–S22

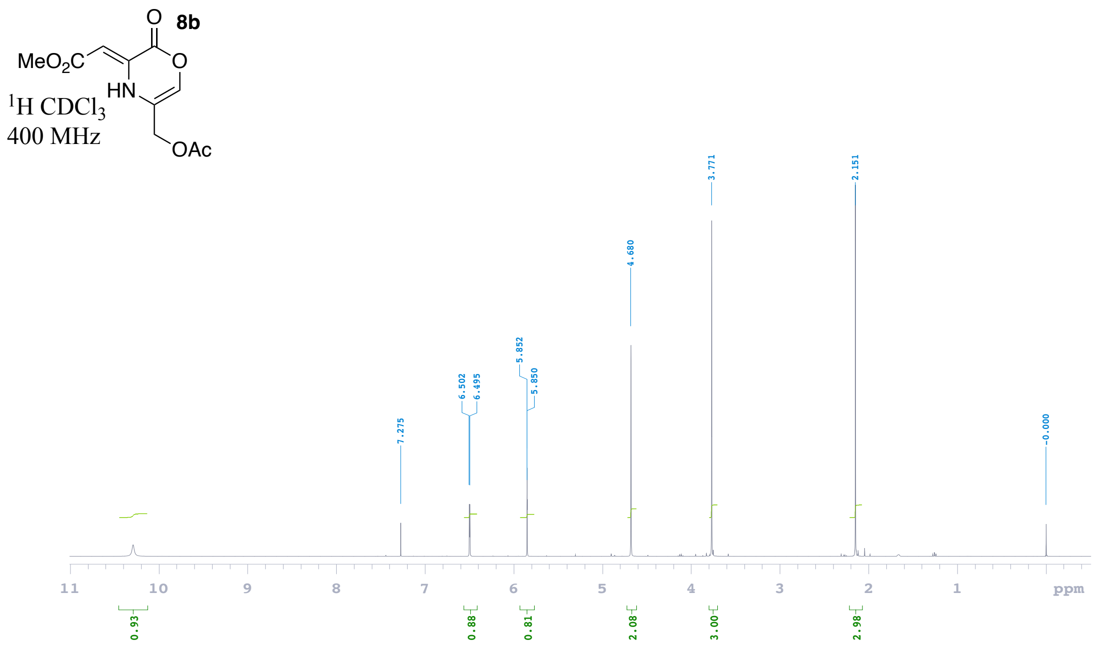
8b
MeO2C
HN
O
O
OAc
1H CDCl3
400 MHz
7.275
6.502
6.495
5.852
5.850
4.680
3.771
2.151
-0.000
0.93
0.88
0.81
2.08
3.00
2.98
ppm

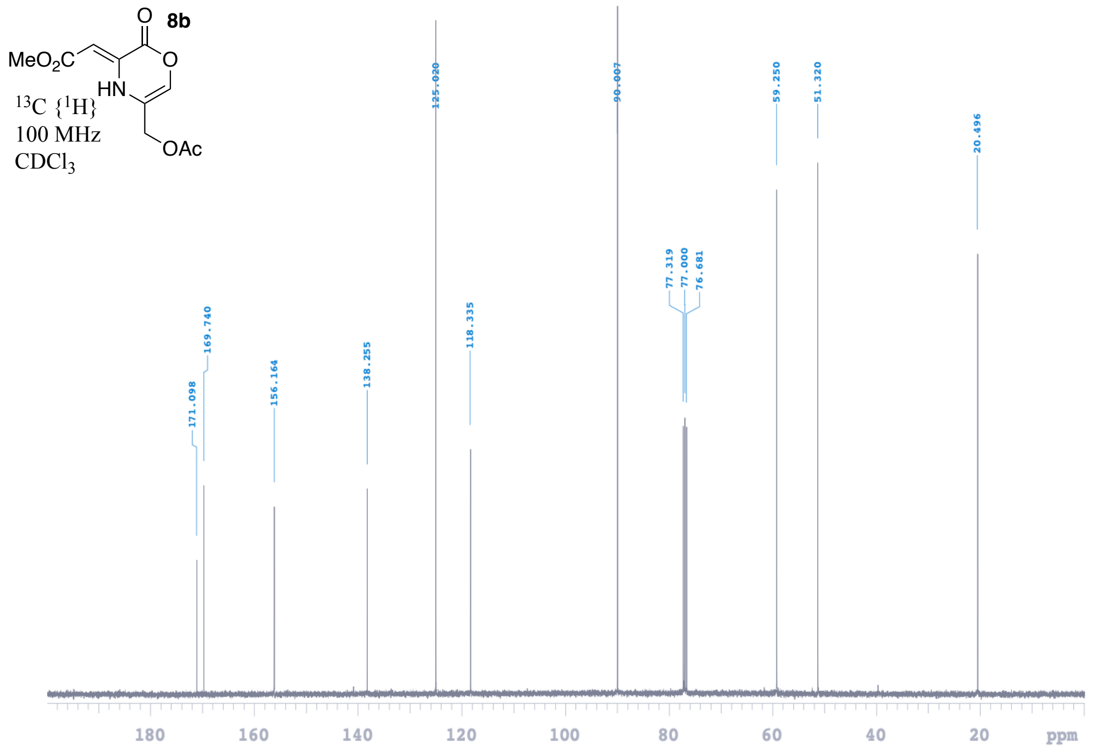
8b
MeO2C
HN
O
O
OAc
13C {1H}
100 MHz
CDCl3
171.098
169.740
156.164
138.255
125.020
118.335
90.007
77.319
77.000
76.681
59.250
51.320
20.496
ppm

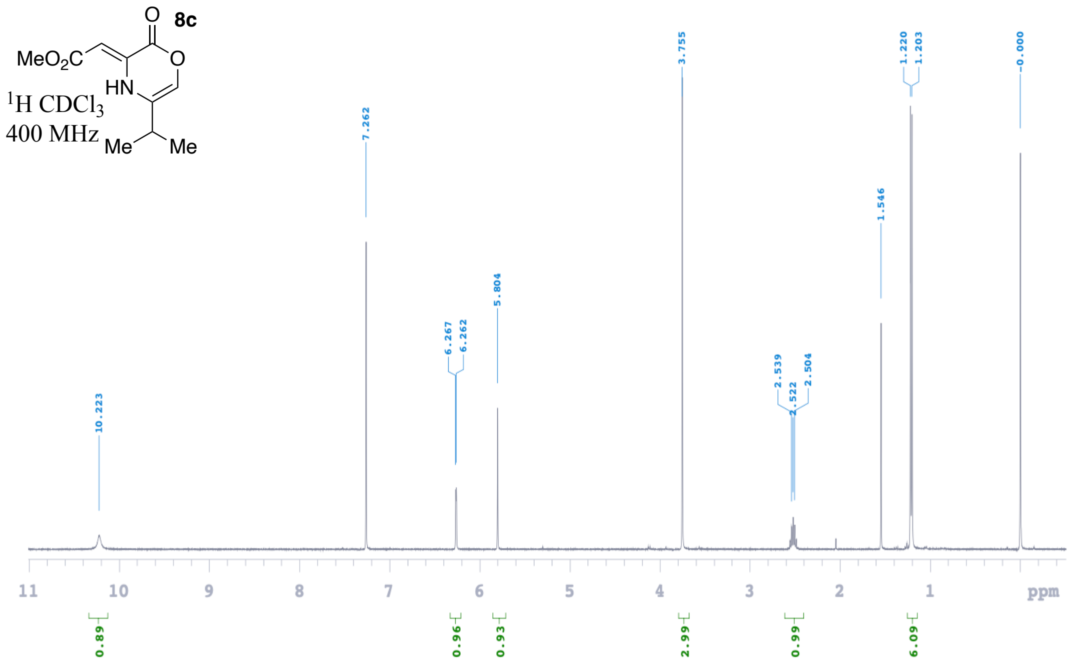

8c
MeO2C
HN
O
Me
Me
1H CDCl3
400 MHz
10.223
7.262
6.267
6.262
5.804
3.755
2.539
2.522
2.504
1.546
1.220
1.203
0.000
ppm
0.89
0.96
0.93
2.99
0.99
6.09

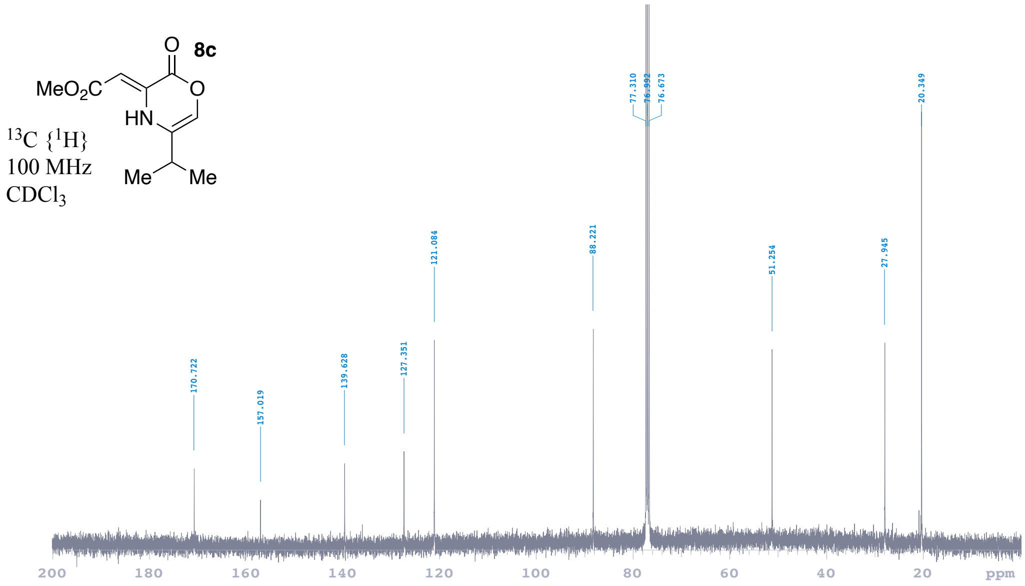

8c
MeO2C
HN
O
Me
Me
13C {1H}
100 MHz
CDCl3
170.722
157.019
139.628
127.351
121.084
88.221
77.310
76.992
76.673
51.254
27.945
20.349
ppm

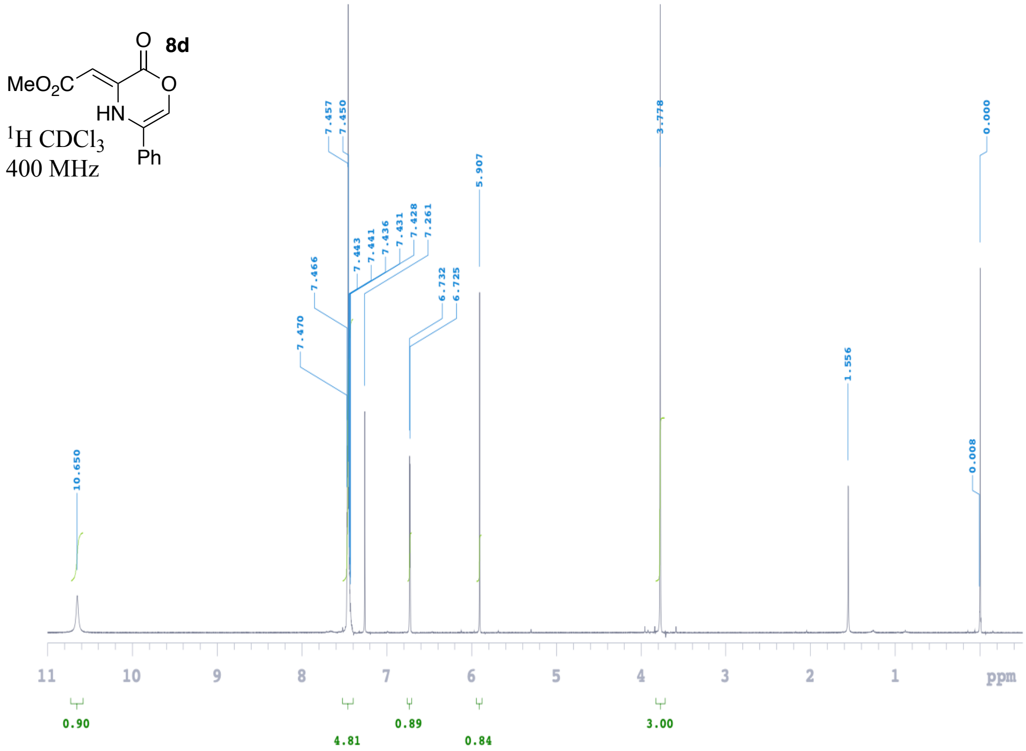
8d
MeO2C
HN
O
O
Ph
1H CDCl3
400 MHz
10.650
7.470
7.466
7.457
7.450
7.443
7.441
7.436
7.431
7.428
7.261
6.732
6.725
5.907
3.778
1.556
0.008
0.000
ppm
0.90
4.81
0.89
0.84
3.00

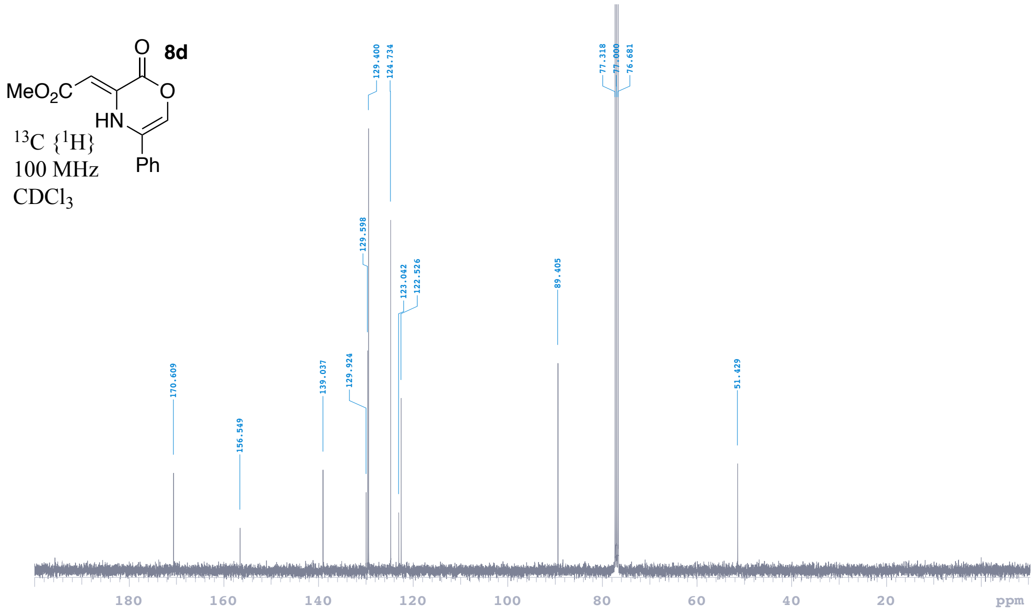
8d
MeO2C
HN
O
O
Ph
13C {1H}
100 MHz
CDCl3
170.609
156.549
139.037
129.924
129.598
129.400
124.734
123.042
122.526
89.405
77.318
77.000
76.681
51.429
ppm

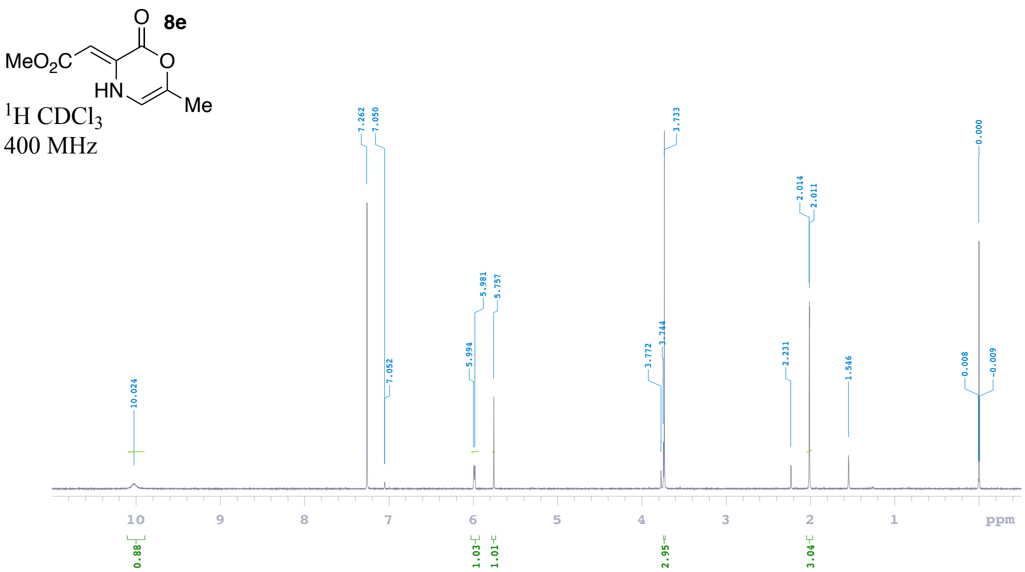

8e
MeO2C
HN
O
O
Me
1H CDCl3
400 MHz
ppm

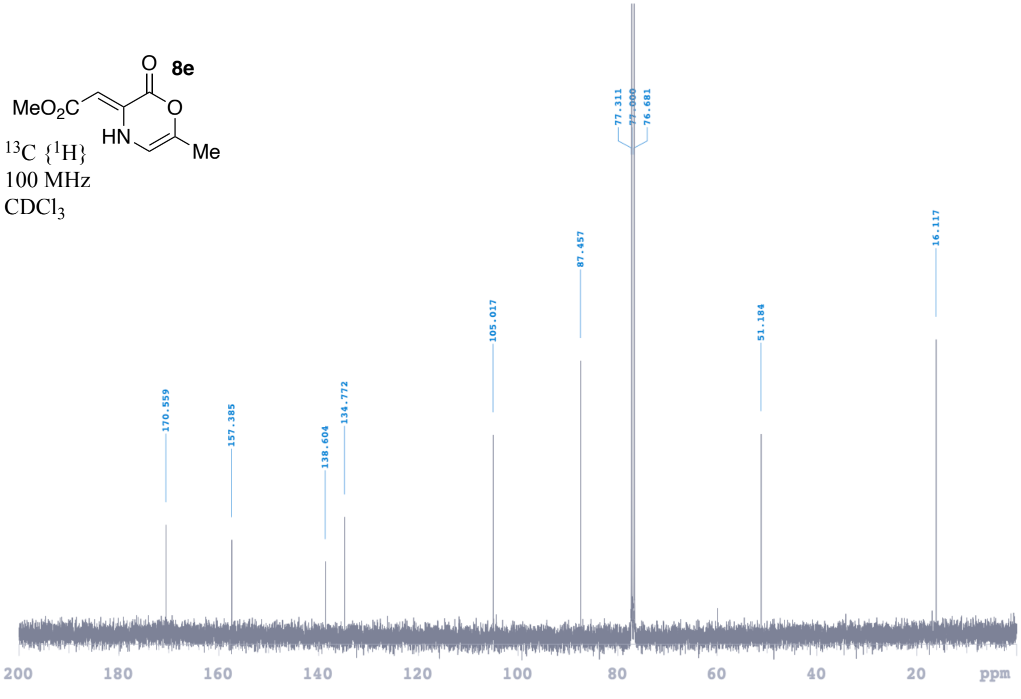

8e
MeO2C
HN
O
O
Me
13C {1H}
100 MHz
CDCl3
ppm

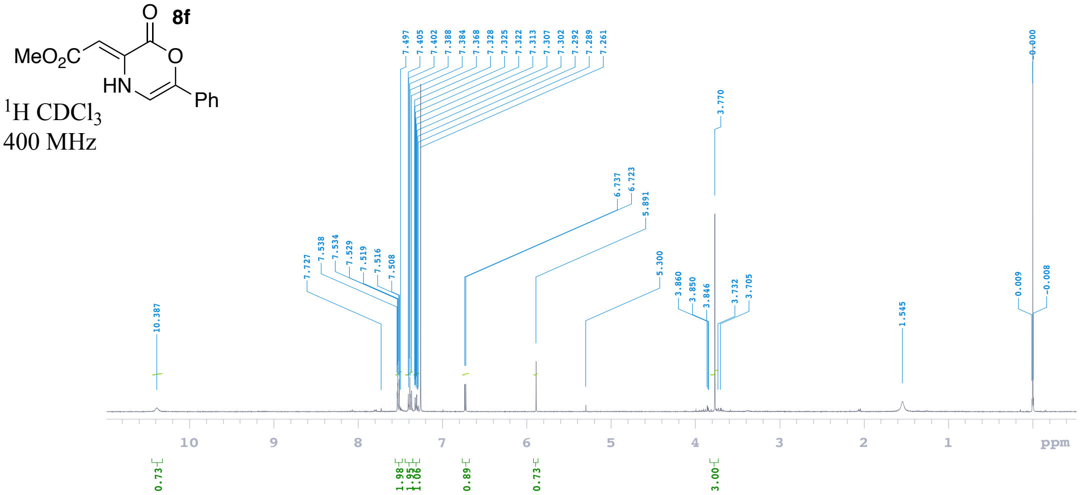
8f
MeO2C
O
HN
Ph
1H CDCl3
400 MHz
10.387
7.727 7.538 7.534 7.529 7.519 7.516 7.508
7.497 7.405 7.402 7.388 7.384 7.368 7.328 7.325 7.322 7.313 7.307 7.302 7.292 7.289 7.261
6.737 6.723
5.891
5.300
3.860 3.850 3.846
3.770
3.732 3.705
1.545
0.009 0.000 -0.008
10 9 8 7 6 5 4 3 2 1 ppm
0.73 1.98 1.95 1.06 0.89 0.73 3.00

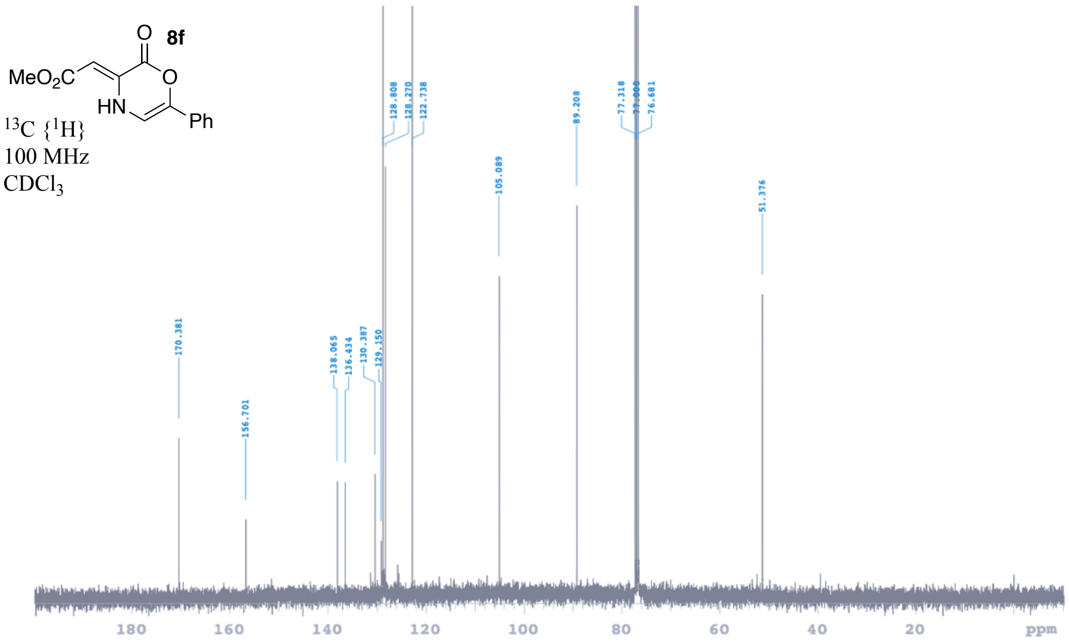
8f
MeO2C
O
HN
Ph
13C {1H}
100 MHz
CDCl3
170.381
156.701
138.065 136.434 130.387 129.150
128.808 128.270 122.738
105.089
89.208
77.318 77.000 76.681
51.376
180 160 140 120 100 80 60 40 20 ppm

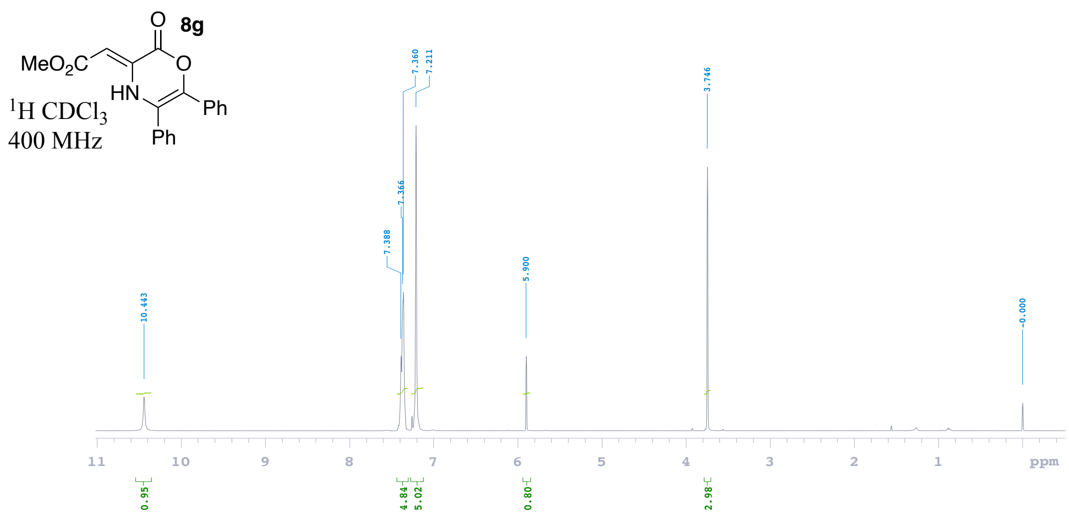
8g
MeO2C
O
HN
Ph
Ph
1H CDCl3
400 MHz
10.443
7.388
7.366
7.360
7.211
5.900
3.746
0.000
0.95
4.84
5.02
0.80
2.98
11
10
9
8
7
6
5
4
3
2
1
ppm

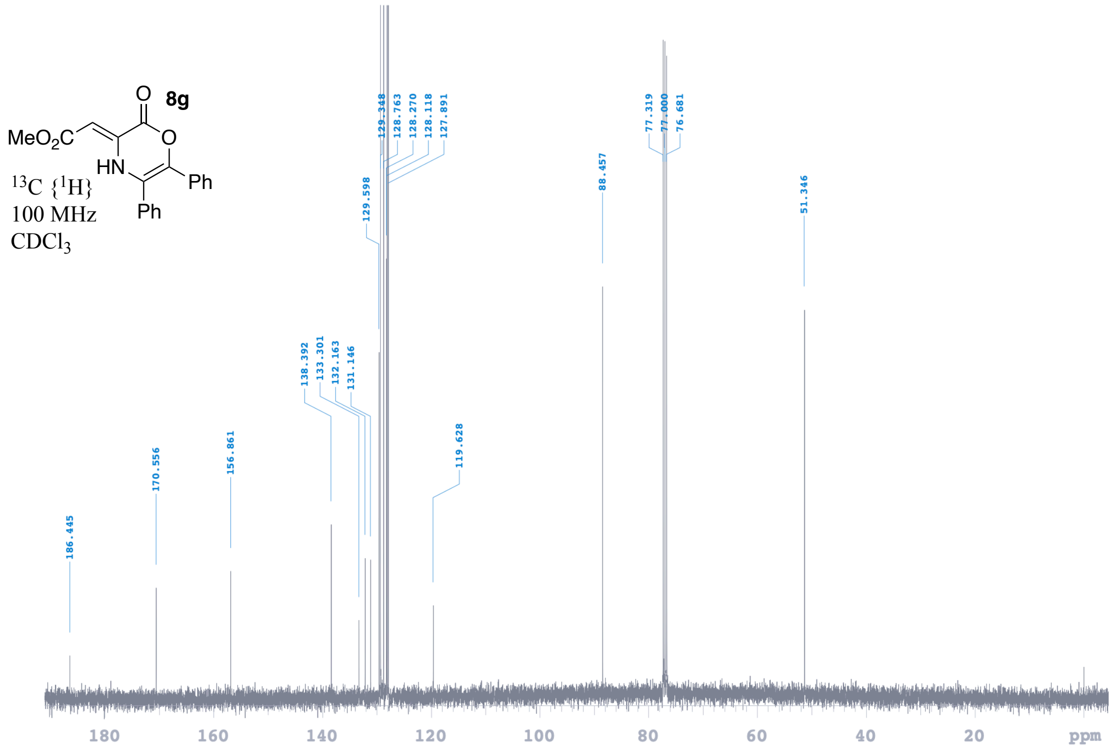
8g
MeO2C
O
HN
Ph
Ph
13C {1H}
100 MHz
CDCl3
186.445
170.556
156.861
138.392
133.301
132.163
131.146
129.598
129.348
128.763
128.270
128.118
127.891
119.628
88.457
77.319
77.000
76.681
51.346
180
160
140
120
100
80
60
40
20
ppm

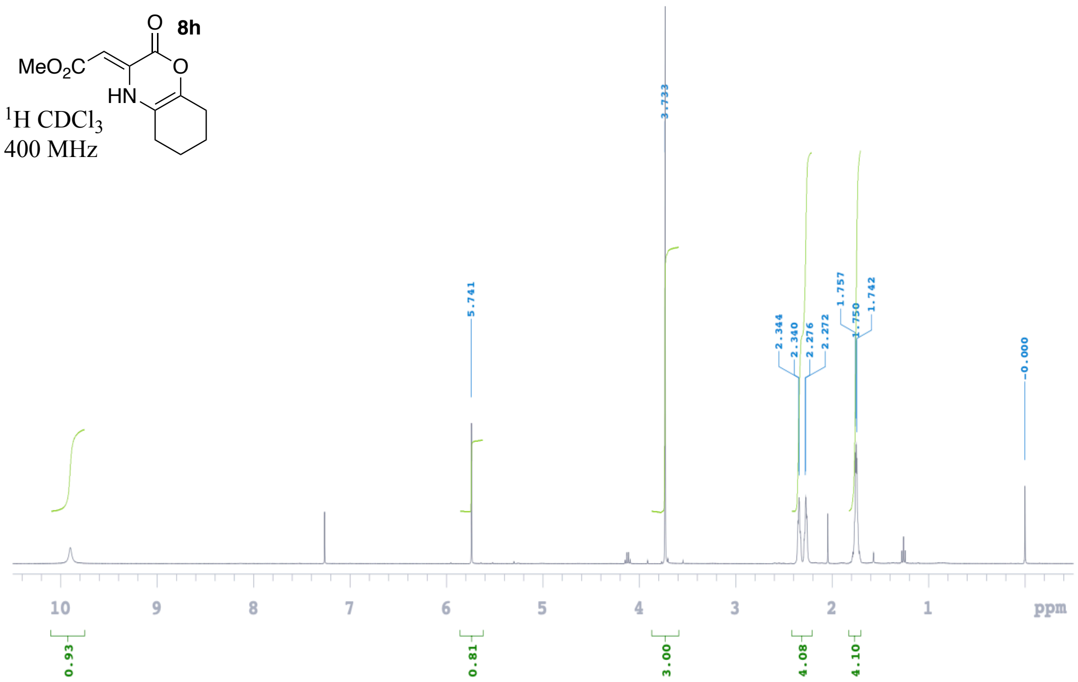
8h
MeO2C
HN
O
^{1}H CDCl$_3$
400 MHz
5.741
3.733
2.344
2.340
2.276
2.272
1.757
1.750
1.742
0.000
10
9
8
7
6
5
4
3
2
1
ppm
0.93
0.81
3.00
4.08
4.10

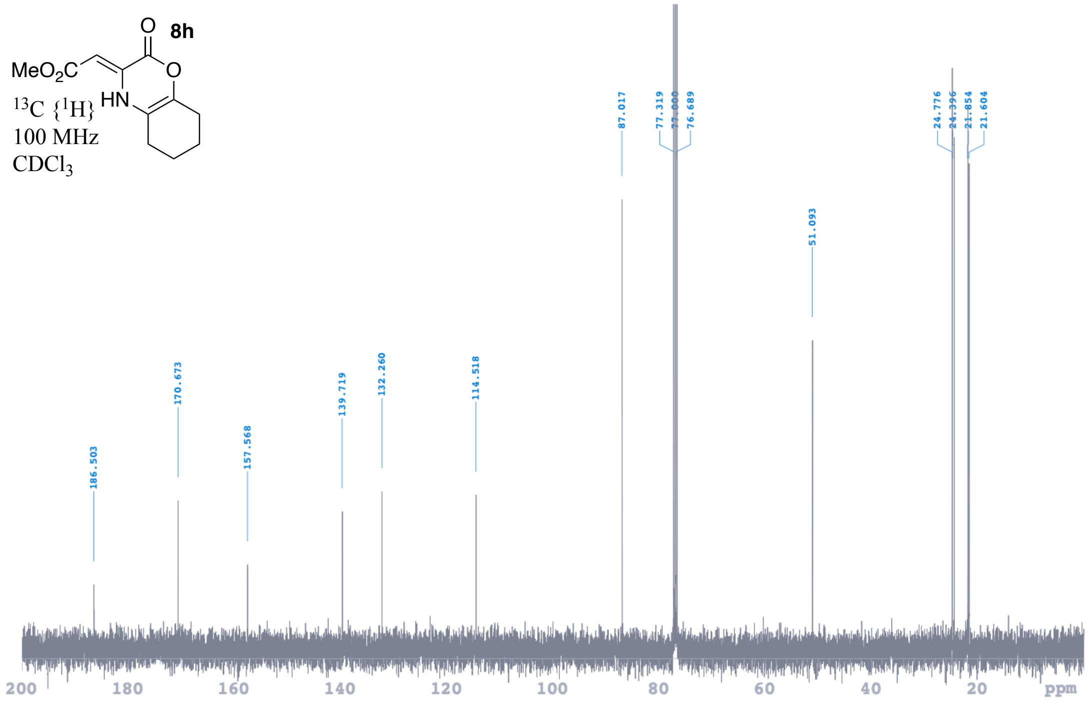
8h
MeO2C
HN
O
^{13}C {^{1}H}
100 MHz
CDCl$_3$
186.503
170.673
157.568
139.719
132.260
114.518
87.017
77.319
76.689
51.093
24.776
21.854
21.604
200
180
160
140
120
100
80
60
40
20
ppm

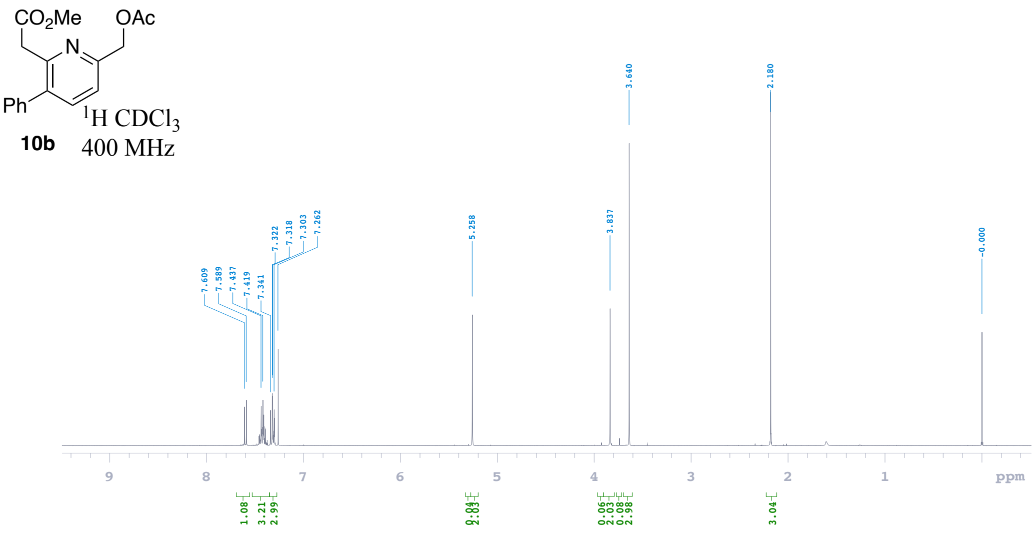
CO2Me
OAc
N
Ph
10b
1H CDCl3
400 MHz
ppm

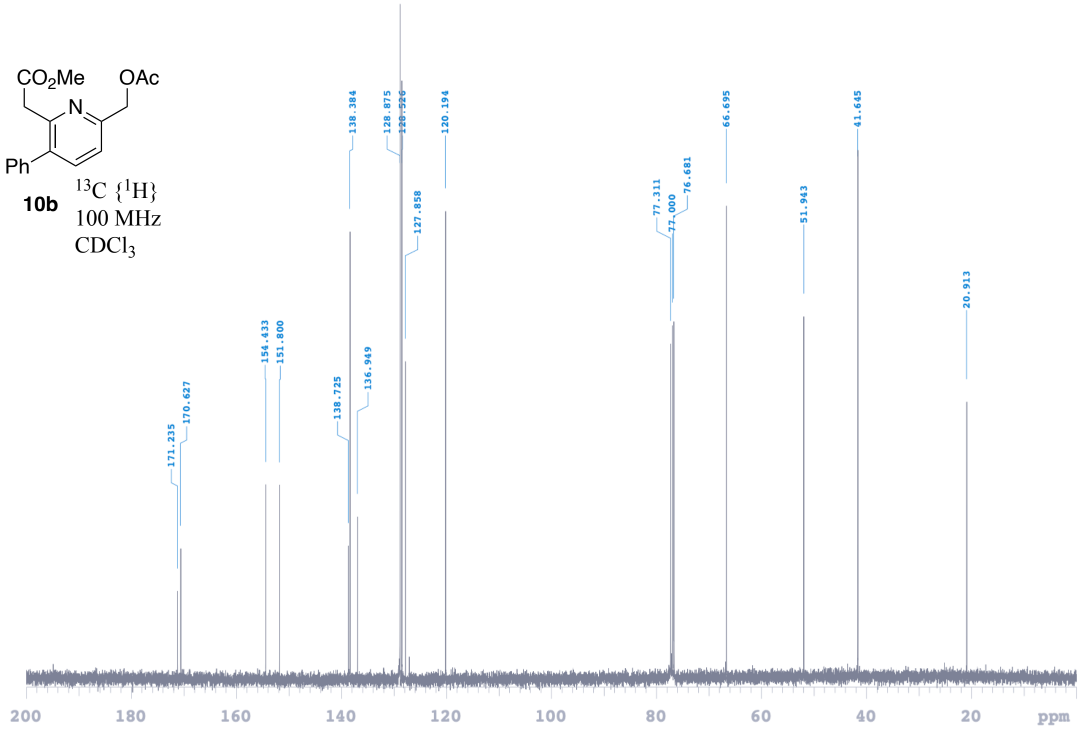
CO2Me
OAc
N
Ph
10b
13C {1H}
100 MHz
CDCl3
ppm

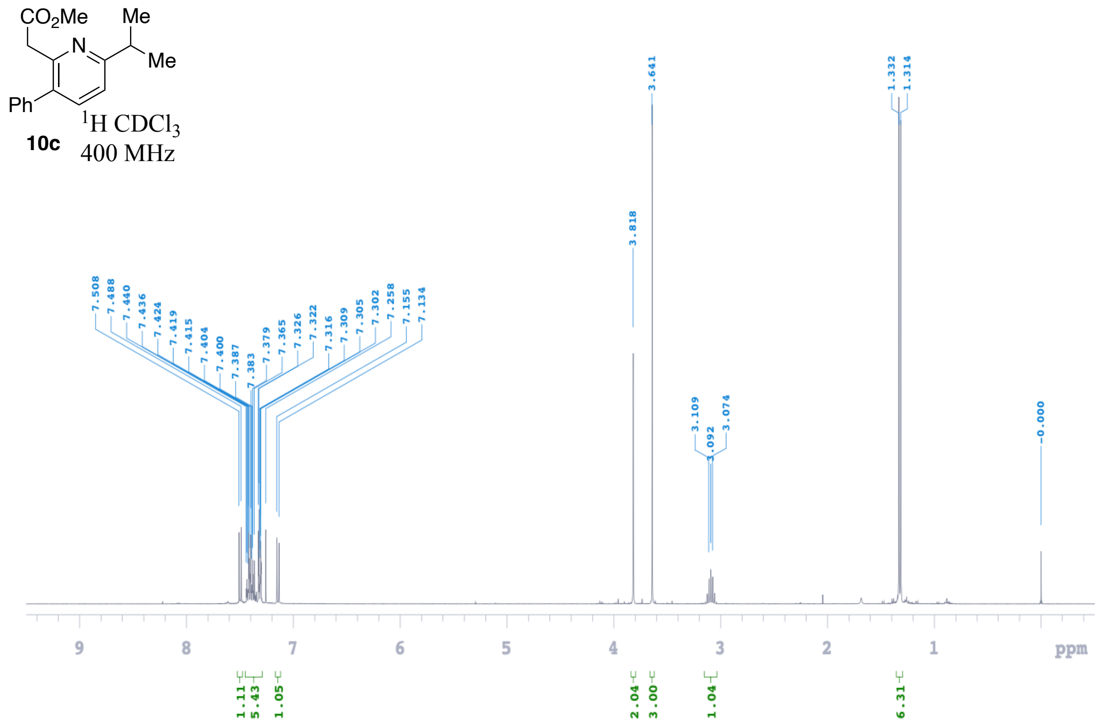
CO2Me
Me
N
Me
Ph
10c
1H CDCl3
400 MHz
7.508 7.488 7.440 7.436 7.424 7.419 7.415 7.404 7.400 7.387 7.383 7.379 7.365 7.326 7.322 7.316 7.309 7.305 7.302 7.258 7.155 7.134
3.818
3.641
3.109 3.092 3.074
1.332 1.314
-0.000
9 8 7 6 5 4 3 2 1 ppm
1.11 5.43 1.05 2.04 3.00 1.04 6.31

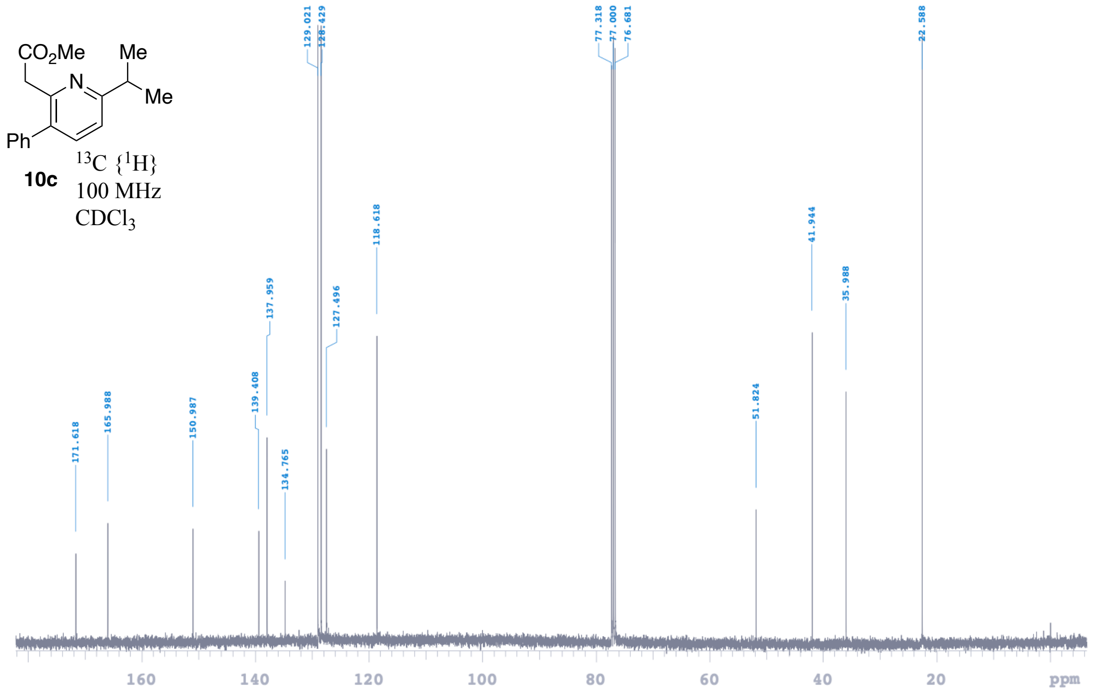
CO2Me
Me
N
Me
Ph
10c
13C {1H}
100 MHz
CDCl3
171.618 165.988 150.987 139.408 137.959 134.765 129.021 128.429 127.496 118.618
77.318 77.000 76.681
51.824 41.944 35.988 22.588
160 140 120 100 80 60 40 20 ppm

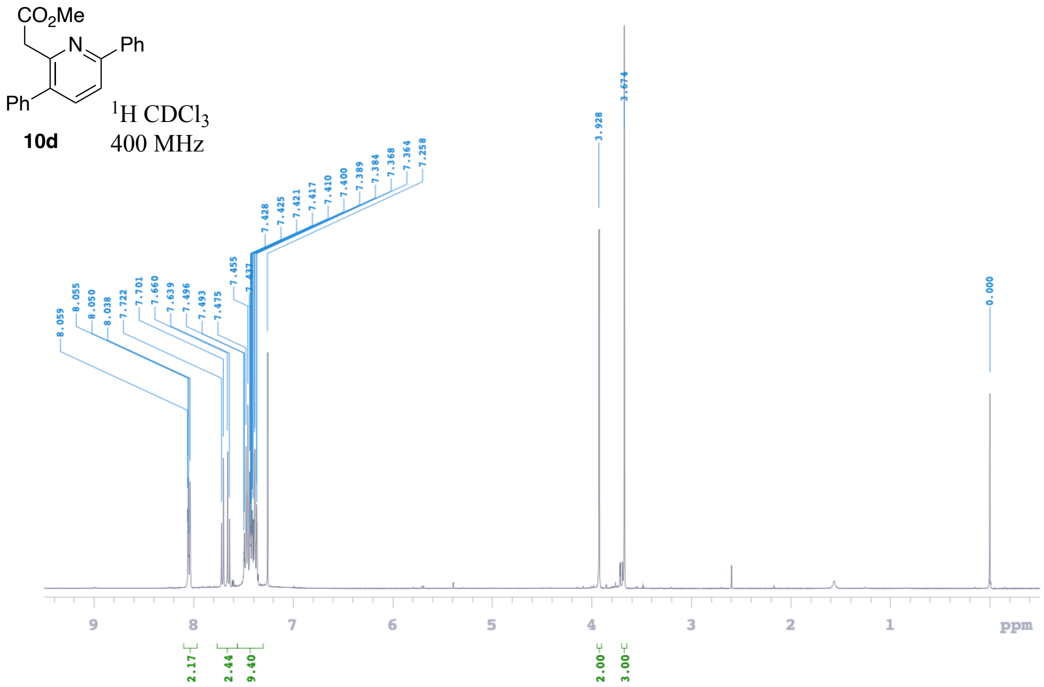
CO2Me
N
Ph
Ph
10d
1H CDCl3
400 MHz
8.059
8.055
8.050
8.038
7.722
7.701
7.660
7.639
7.496
7.493
7.475
7.455
7.437
7.428
7.425
7.421
7.417
7.410
7.400
7.389
7.384
7.368
7.364
7.258
3.928
3.674
0.000
9
8
7
6
5
4
3
2
1
ppm
2.17
2.44
9.40
2.00
3.00

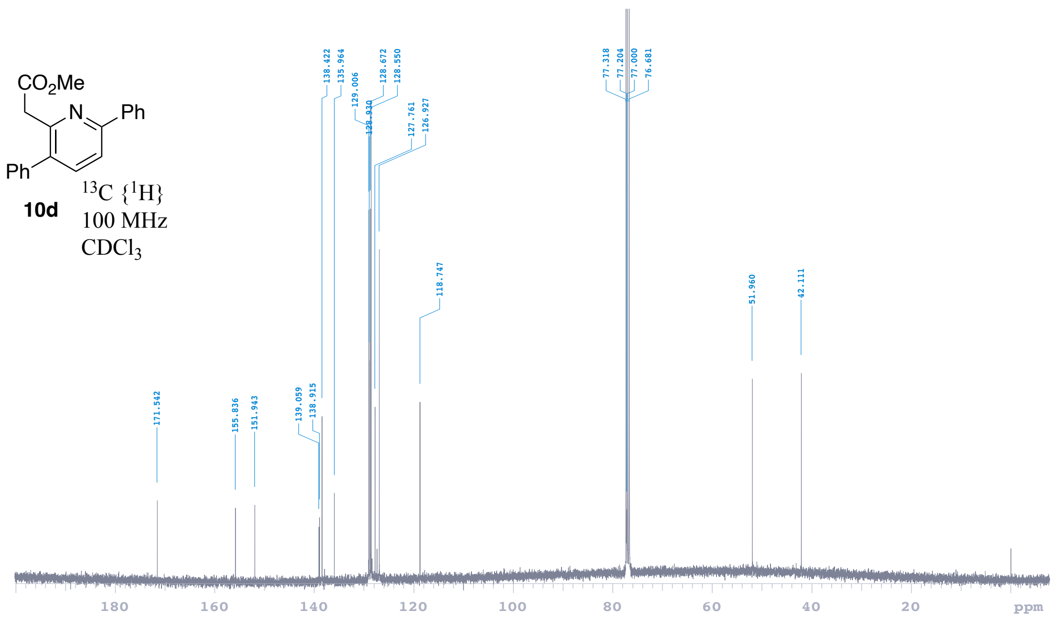
CO2Me
N
Ph
Ph
10d
13C {1H}
100 MHz
CDCl3
171.542
155.836
151.943
139.059
138.915
138.422
135.964
129.006
128.930
128.672
128.550
127.761
126.927
118.747
77.318
77.204
77.000
76.681
51.960
42.111
180
160
140
120
100
80
60
40
20
ppm

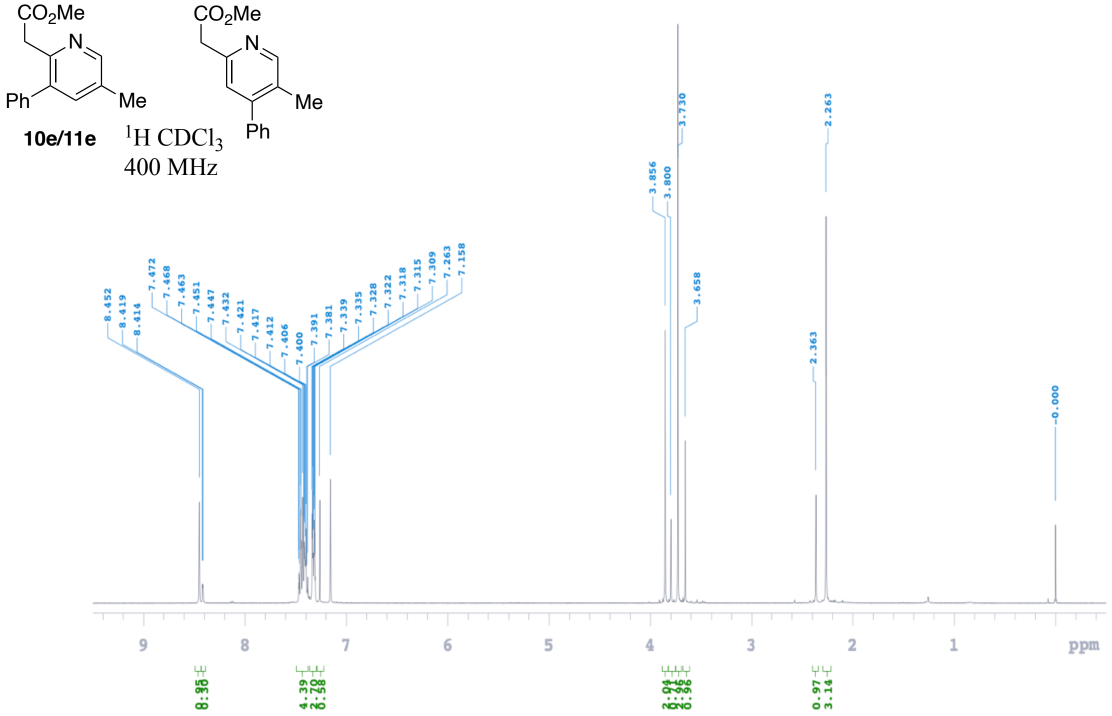
CO2Me
N
Ph
Me
10e/11e
1H CDCl3
400 MHz
8.452
8.419
8.414
7.472
7.468
7.463
7.451
7.447
7.432
7.421
7.417
7.412
7.406
7.400
7.391
7.381
7.339
7.335
7.328
7.322
7.318
7.315
7.309
7.263
7.158
3.856
3.800
3.730
3.658
2.363
2.263
0.000
9
8
7
6
5
4
3
2
1
ppm
4.39
0.97
3.14

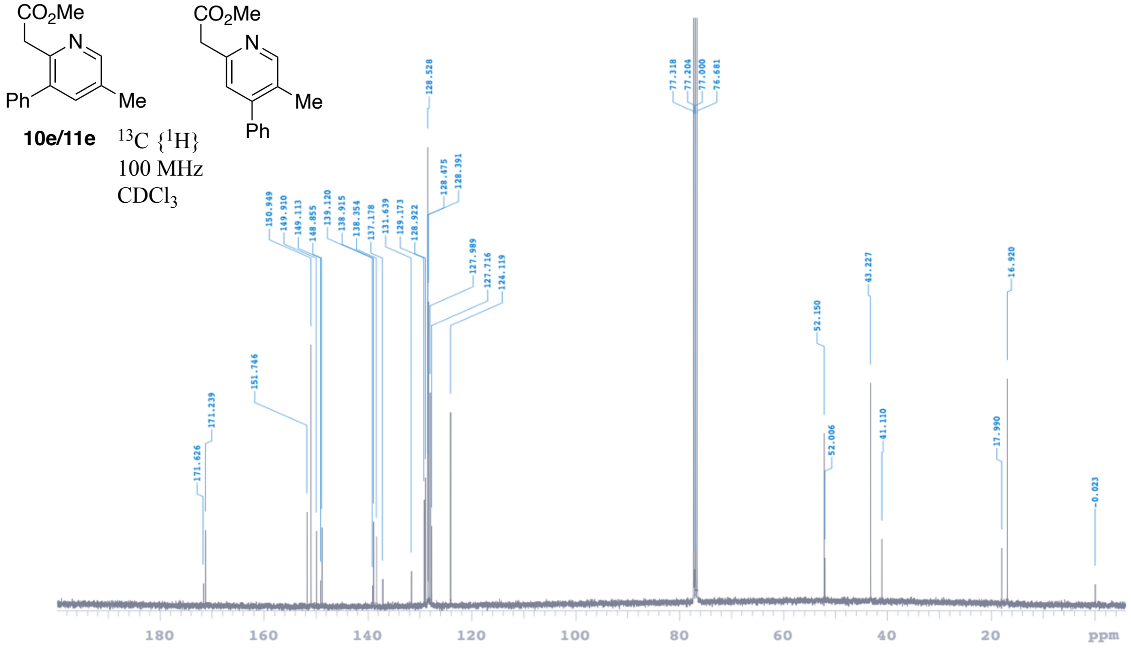
CO2Me
N
Ph
Me
10e/11e
13C {1H}
100 MHz
CDCl3
171.626
171.239
151.746
150.949
149.910
149.113
148.855
139.120
138.915
138.354
137.178
131.639
129.173
128.922
128.528
128.475
128.391
127.989
127.716
124.119
77.318
77.204
77.000
76.681
52.150
52.006
43.227
41.110
17.990
16.920
0.023
180
160
140
120
100
80
60
40
20
ppm

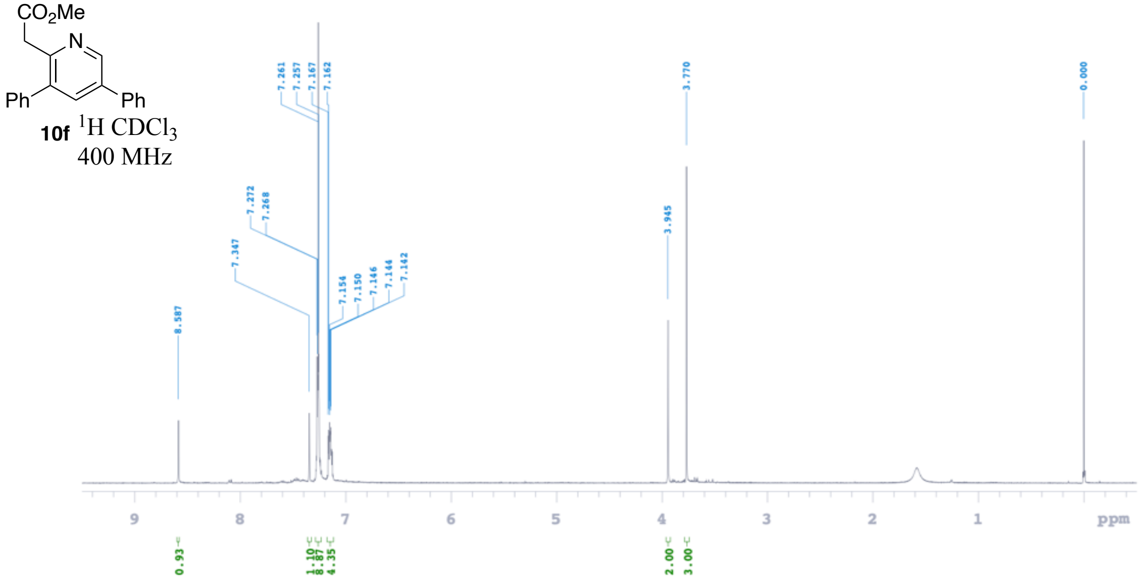
CO2Me
N
Ph
Ph
10f
1H CDCl3
400 MHz
8.587
7.347
7.272
7.268
7.261
7.257
7.167
7.162
7.154
7.150
7.146
7.144
7.142
3.945
3.770
0.000
9
8
7
6
5
4
3
2
1
ppm
0.93
1.10
8.87
4.35
2.00
3.00

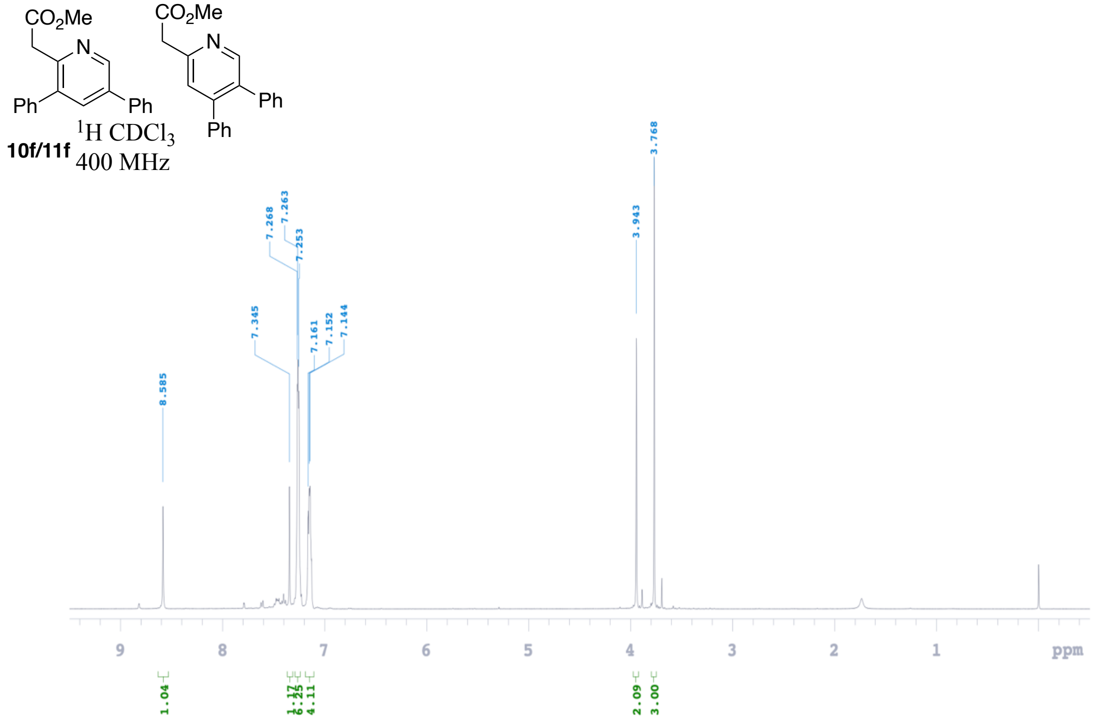
CO2Me
N
Ph
Ph
CO2Me
N
Ph
Ph
1H CDCl3
10f/11f
400 MHz
8.585
7.345
7.268
7.263
7.253
7.161
7.152
7.144
3.943
3.768
9
8
7
6
5
4
3
2
1
ppm
1.04
2.09
3.00
4.11

–

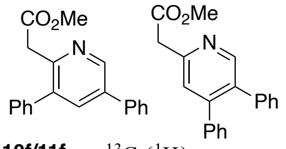

10f/11f

$^{13}C\ \{^{1}H\}$
100 MHz
$CDCl_3$

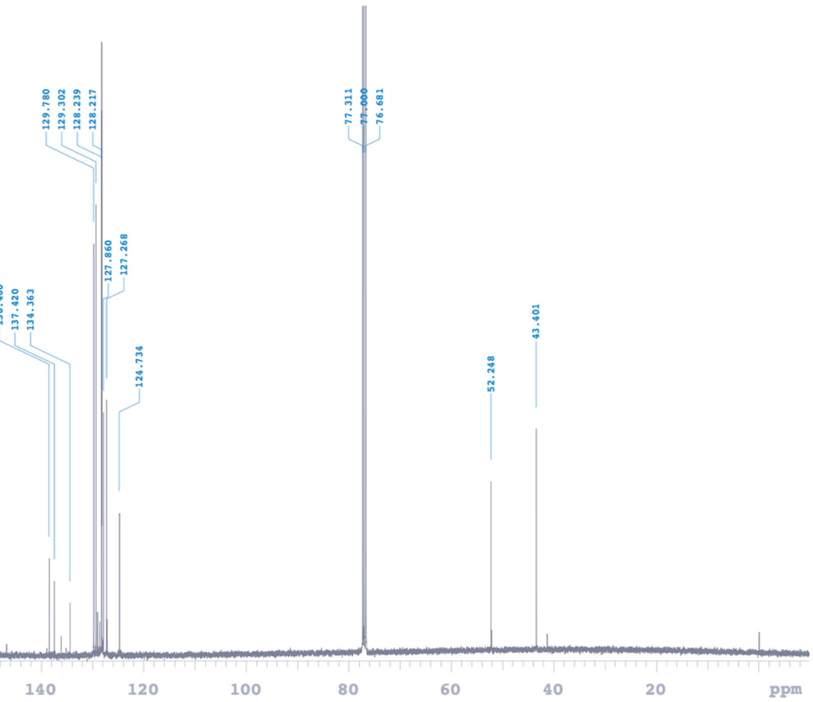

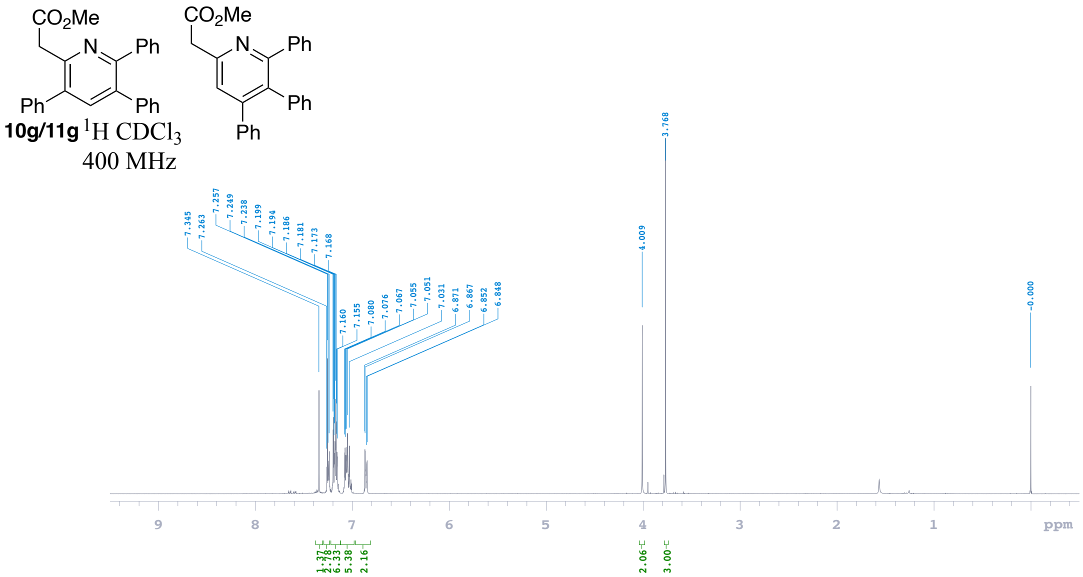
CO2Me
N
Ph
Ph
Ph
10g/11g 1H CDCl3
400 MHz
CO2Me
N
Ph
Ph
Ph
ppm

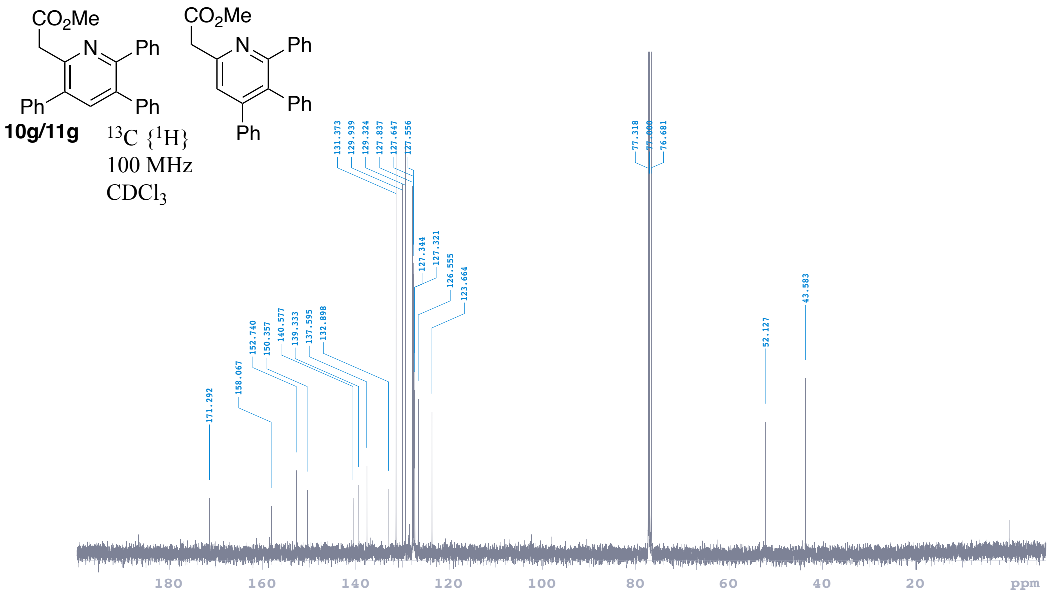
CO2Me
N
Ph
Ph
Ph
10g/11g
13C {1H}
100 MHz
CDCl3
CO2Me
N
Ph
Ph
Ph
ppm

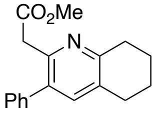

^1H $CDCl_3$ **10h**
400 MHz

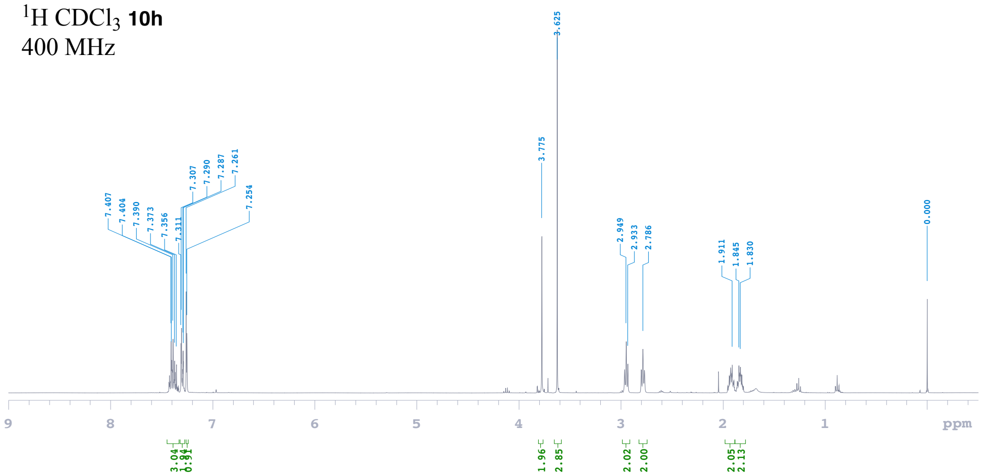

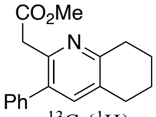

^{13}C {^1H} **10h**
100 MHz
$CDCl_3$

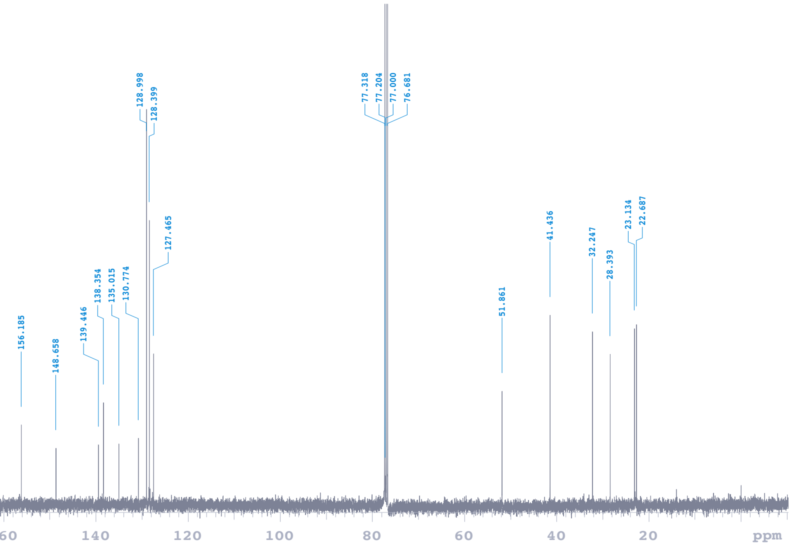

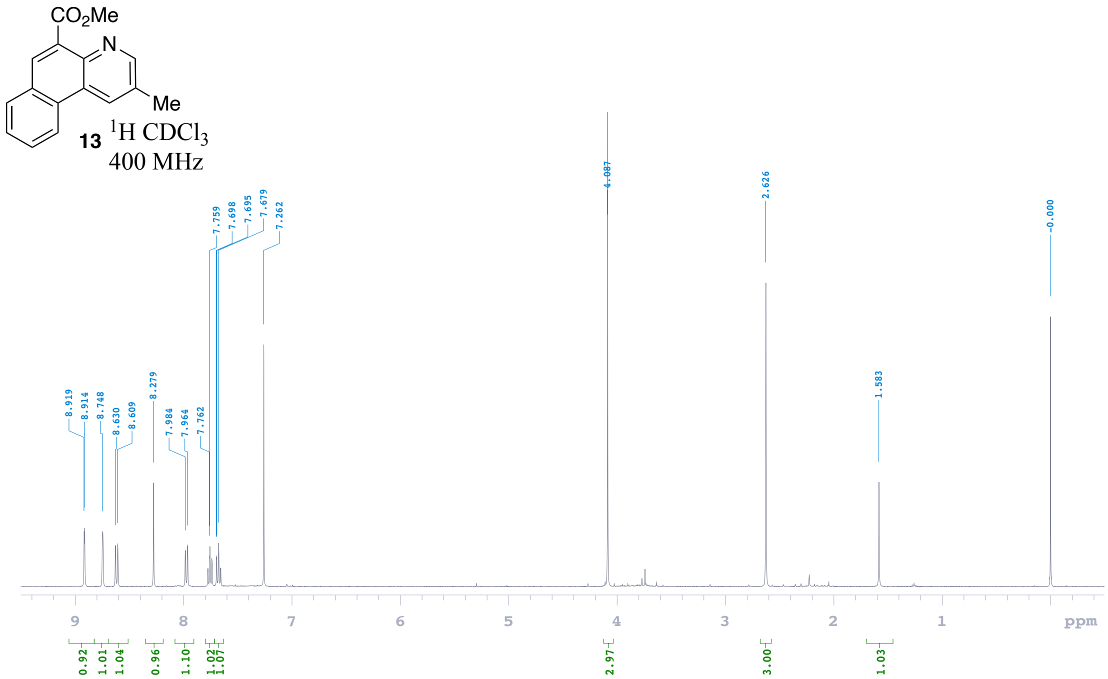
CO2Me
N
Me
13 1H CDCl3
400 MHz
8.919
8.914
8.748
8.630
8.609
8.279
7.984
7.964
7.762
7.759
7.698
7.695
7.679
7.262
4.087
2.626
1.583
0.000
9
8
7
6
5
4
3
2
1
ppm
0.92
1.01
1.04
0.96
1.10
1.02
1.07
2.97
3.00
1.03

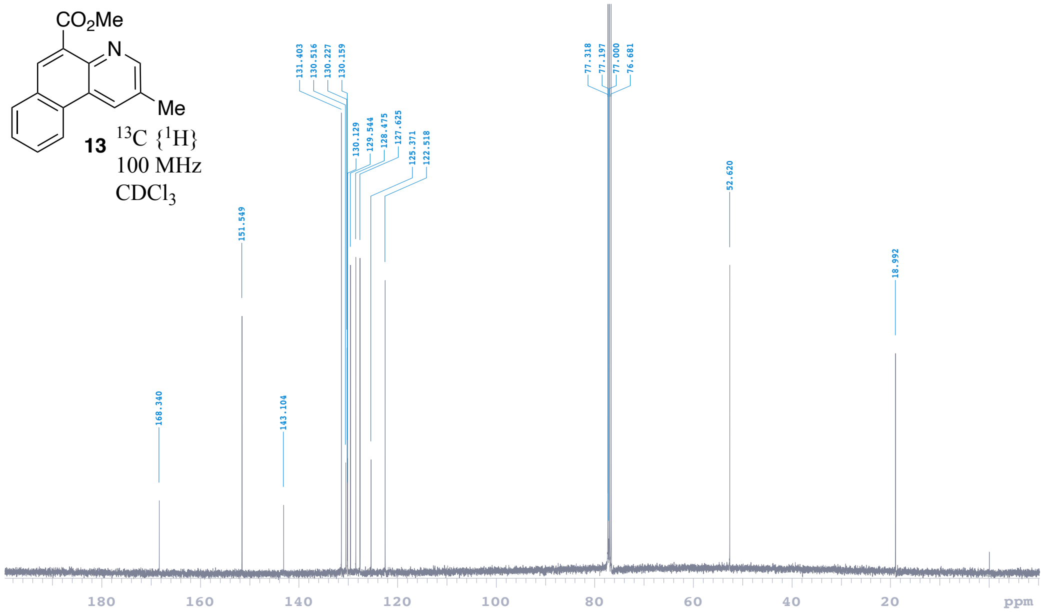
CO2Me
N
Me
13 13C {1H}
100 MHz
CDCl3
168.340
151.549
143.104
131.403
130.516
130.227
130.159
130.129
129.544
128.475
127.625
125.371
122.518
77.318
77.197
77.000
76.681
52.620
18.992
180
160
140
120
100
80
60
40
20
ppm

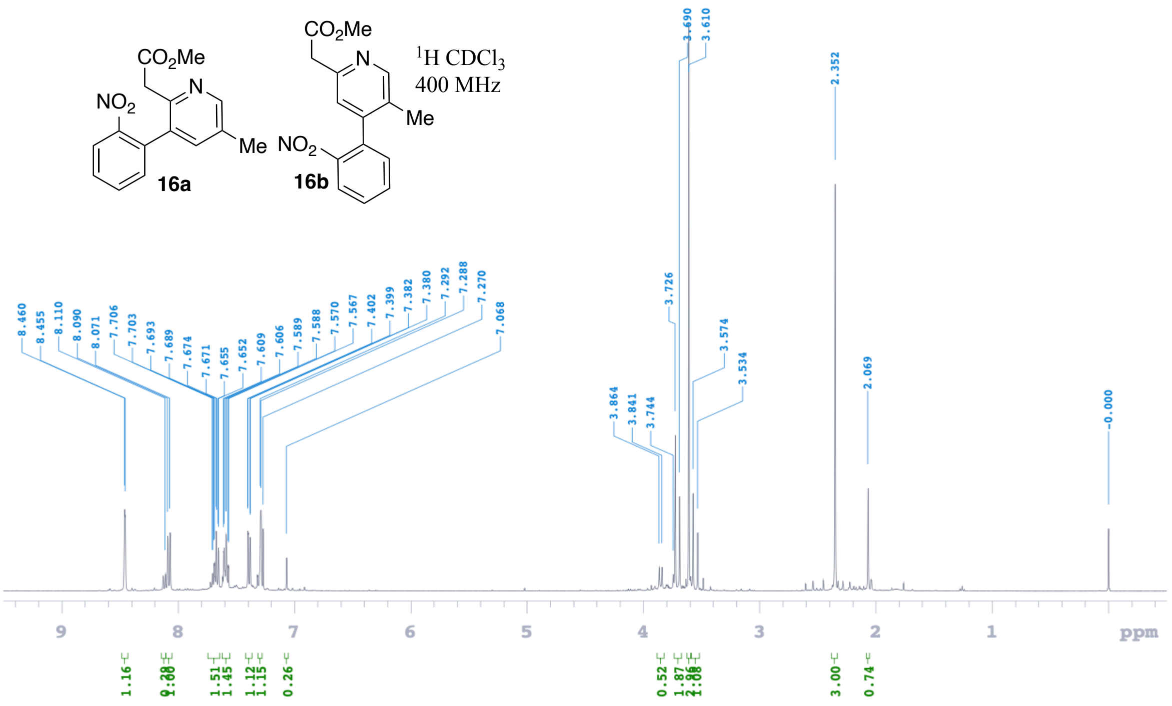

CO2Me
N
NO2
Me
16a
CO2Me
N
Me
NO2
16b
1H CDCl3
400 MHz
8.460
8.455
8.110
8.090
8.071
7.706
7.703
7.693
7.689
7.674
7.671
7.655
7.652
7.609
7.606
7.589
7.588
7.570
7.567
7.402
7.399
7.382
7.380
7.292
7.288
7.270
7.068
3.864
3.841
3.744
3.726
3.690
3.610
3.574
3.534
2.352
2.069
-0.000
9
8
7
6
5
4
3
2
1
ppm
1.16
1.00
1.51
1.45
1.12
1.15
0.26
0.52
1.87
3.00
0.74

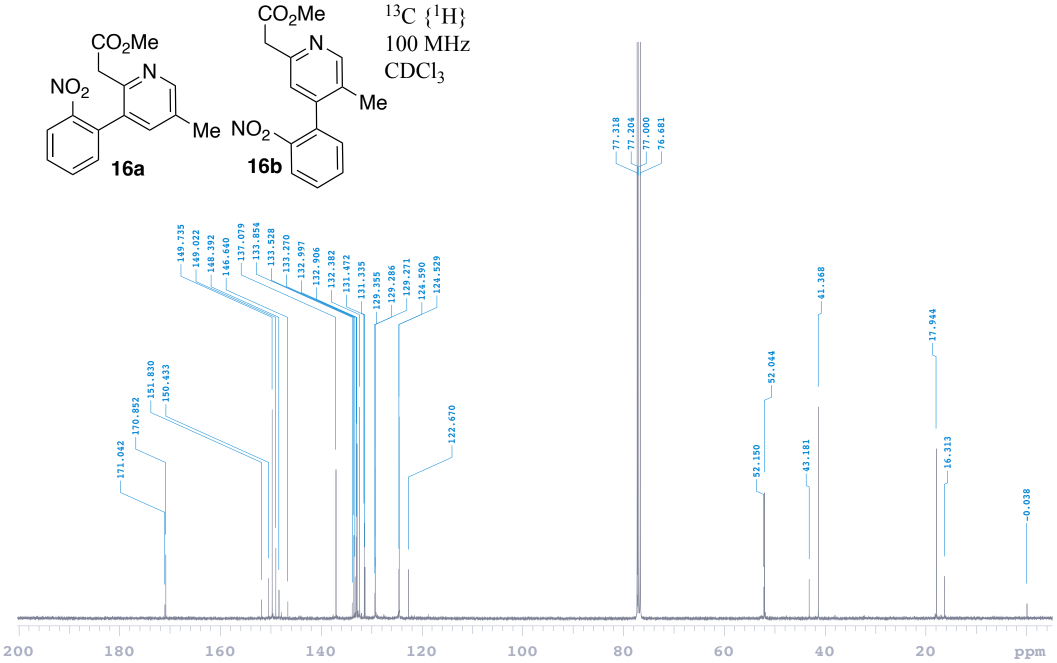

CO2Me
N
NO2
Me
16a
CO2Me
N
Me
NO2
16b
13C {1H}
100 MHz
CDCl3
77.318
77.204
77.000
76.681
171.042
170.852
151.830
150.433
149.735
149.022
148.392
146.640
137.079
133.854
133.528
133.270
132.997
132.906
132.382
131.472
131.335
129.355
129.286
129.271
124.590
124.529
122.670
52.150
52.044
43.181
41.368
17.944
16.313
-0.038
200
180
160
140
120
100
80
60
40
20
ppm

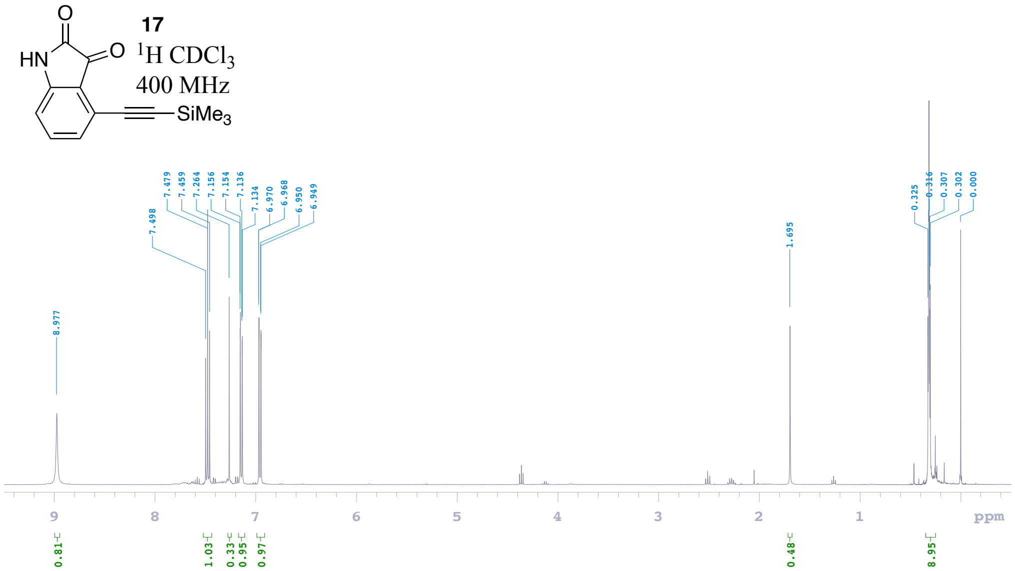
17
^{1}H CDCl$_3$
400 MHz
HN
O
O
SiMe$_3$
ppm

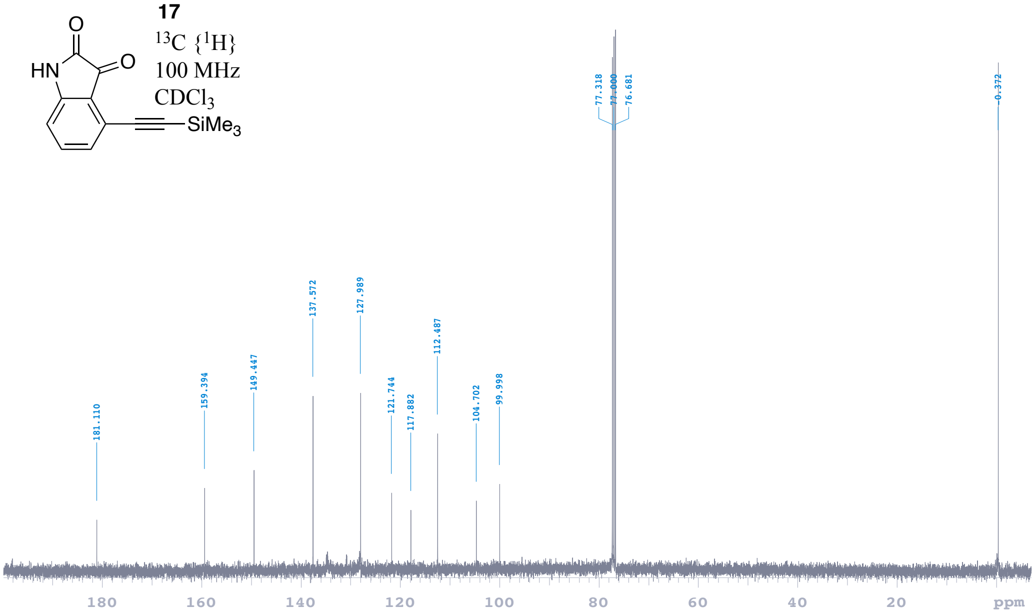
17
^{13}C {^{1}H}
100 MHz
CDCl$_3$
HN
O
O
SiMe$_3$
ppm

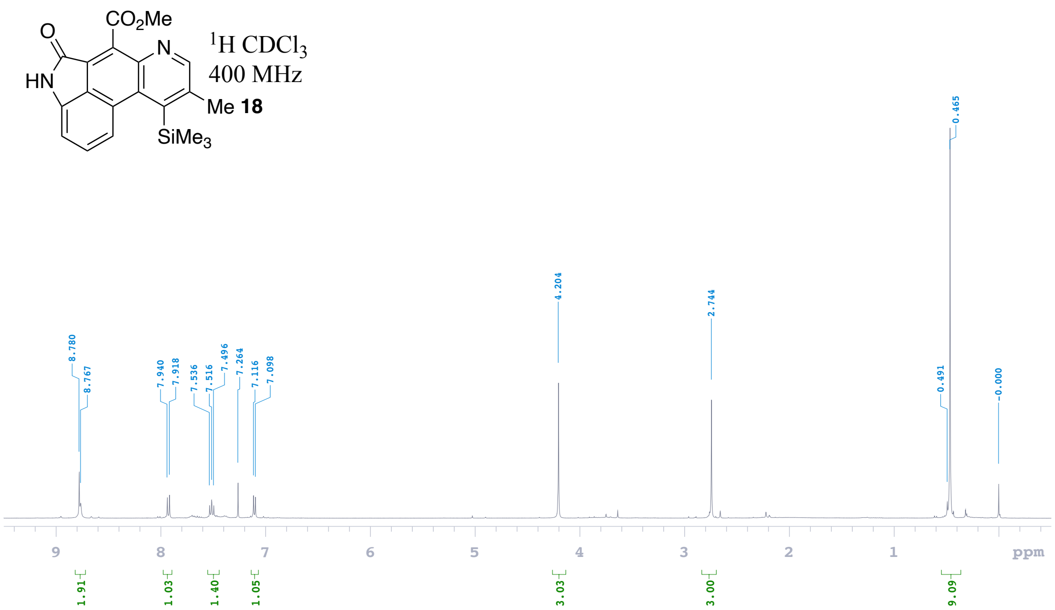
CO2Me
O
N
HN
Me 18
SiMe3
1H CDCl3
400 MHz
8.780
8.767
7.940
7.918
7.536
7.516
7.496
7.264
7.116
7.098
4.204
2.744
0.491
0.465
0.000
9 8 7 6 5 4 3 2 1 ppm
1.91
1.03
1.40
1.05
3.03
3.00
9.09

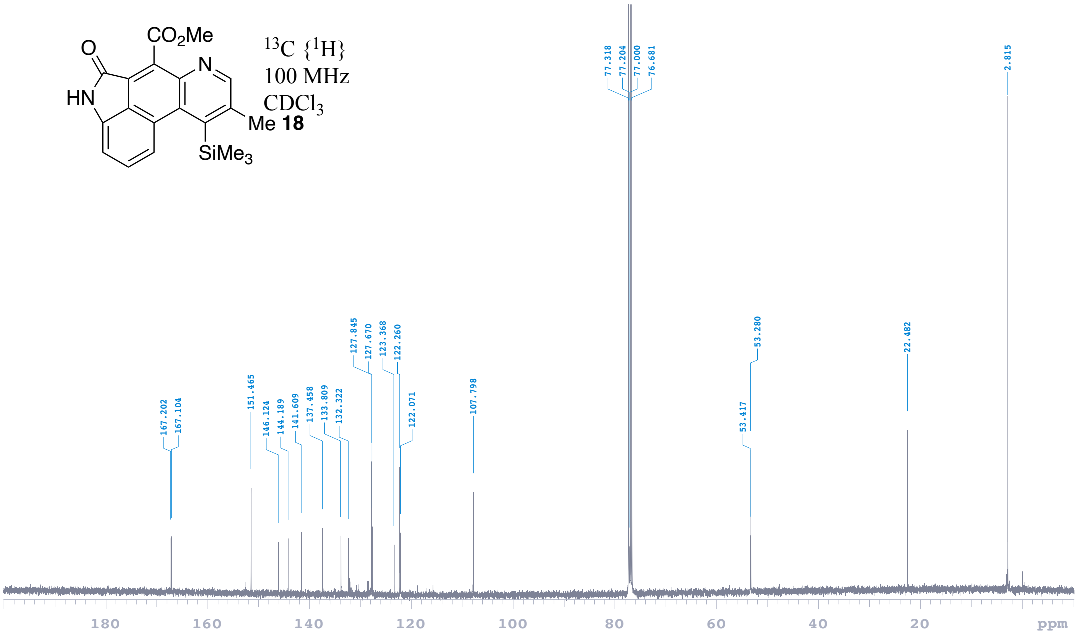
CO2Me
O
N
HN
Me 18
SiMe3
13C {1H}
100 MHz
CDCl3
167.202
167.104
151.465
146.124
144.189
141.609
137.458
133.809
132.322
127.845
127.670
123.368
122.260
122.071
107.798
77.318
77.204
77.000
76.681
53.417
53.280
22.482
2.815
180 160 140 120 100 80 60 40 20 ppm

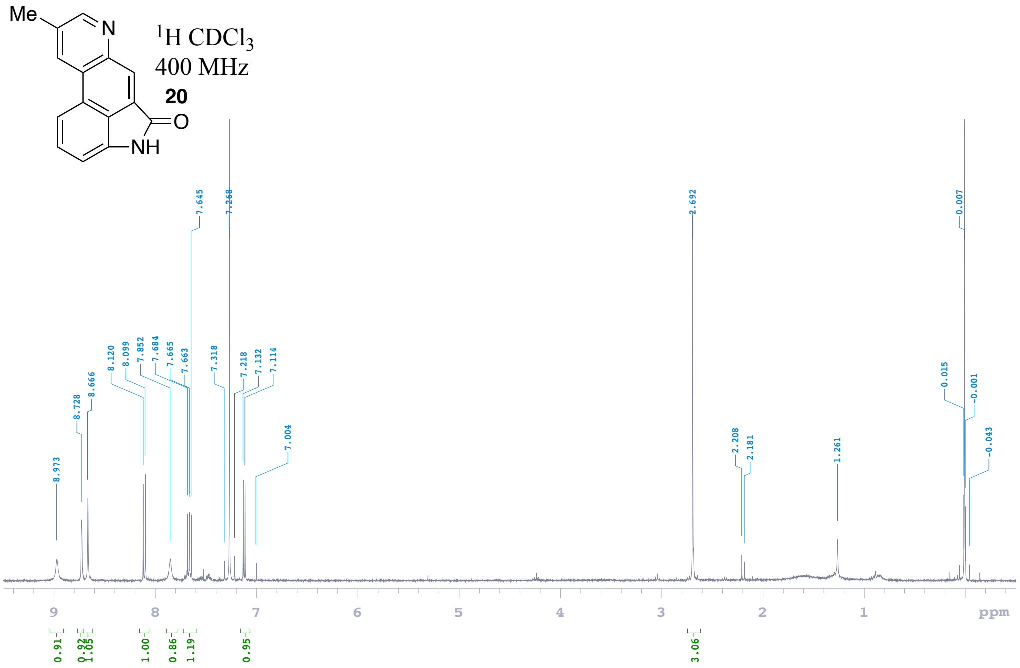
Me
N
O
NH
1H CDCl3
400 MHz
20
ppm

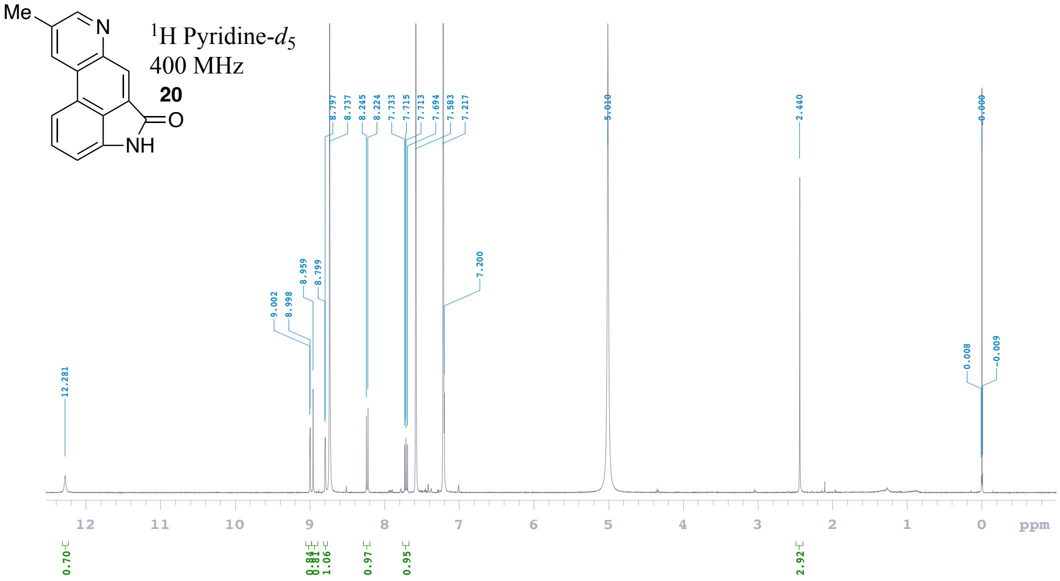
Me
N
O
NH
1H Pyridine-d5
400 MHz
20
ppm

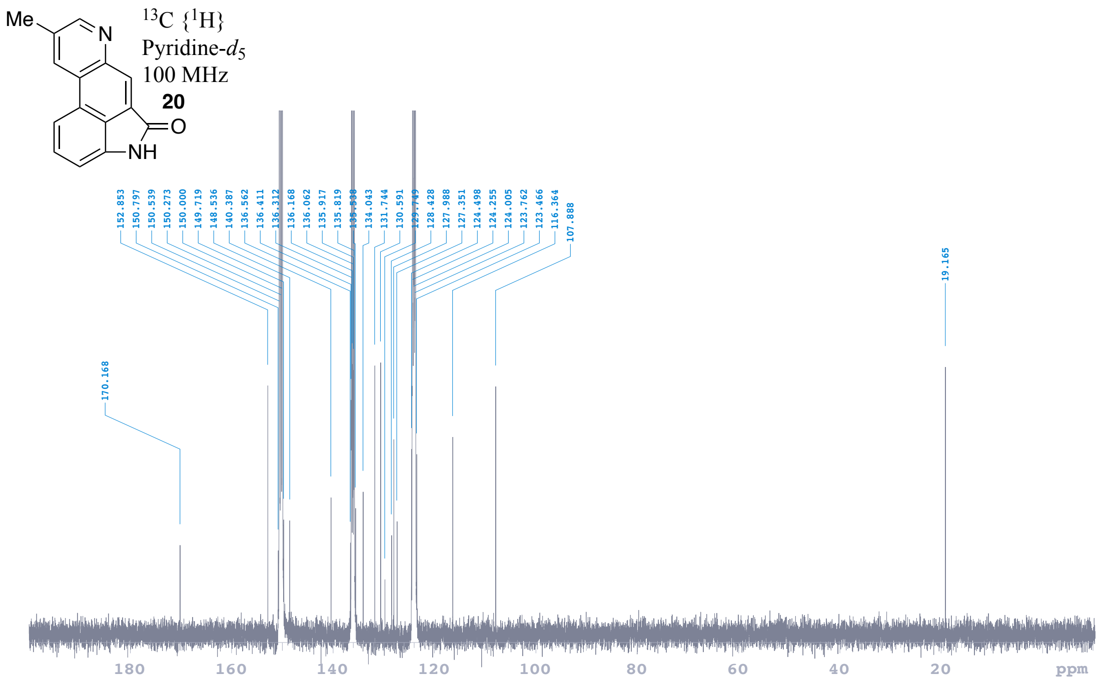
Me
N
O
NH
20
13C {1H}
Pyridine-d5
100 MHz
170.168
152.853
150.797
150.539
150.273
150.000
149.719
148.536
140.387
136.562
136.411
136.312
136.168
136.062
135.917
135.819
134.043
131.744
130.591
128.428
127.988
127.351
124.498
124.255
124.005
123.762
123.466
116.364
107.888
19.165
180
160
140
120
100
80
60
40
20
ppm
